# Uniaxial Compression Constitutive Behavior of Mixed Recycled Brick-Aggregate Concrete After Carbonation

**DOI:** 10.3390/ma19183845

**Published:** 2026-09-10

**Authors:** Qun Zhang, Lin Yao, Xing Zhao, Lifang Zhang, Haiyang Li, Jin Wu

**Affiliations:** 1 Eastern Airport Co., Ltd., Nanjing 211100, China; 2 College of Civil Aviation, Nanjing University of Aeronautics and Astronautics, Nanjing 211106, China

**Keywords:** mixed recycled brick aggregate, brick-aggregate replacement ratio, recycled aggregate concrete, carbonation depth, constitutive relationship

## Abstract

Mixed recycled brick-aggregate concrete (MRBAC), produced by substituting natural coarse aggregates with crushed waste bricks and waste concrete, serves as a promising approach for the resource utilization of construction solid waste and boasts broad engineering application prospects. At present, few studies have established uniaxial compressive constitutive models for MRBAC after accelerated carbonation, which restricts its structural application. In this study, 16 groups of specimens were fabricated with four replacement ratios of recycled brick aggregate (RBA)—0%, 10%, 20%, and 30%—as well as four accelerated carbonation durations—0 d, 40 d, 80 d, and 120 d. Accelerated carbonation tests were carried out to measure carbonation depth, cubic compressive strength, elastic modulus, and uniaxial compressive constitutive curves of MRBAC. Test results show that full 120-day carbonation increases cubic compressive strength and elastic modulus of MRBAC by 16.07–32.13% and 28.3–50.1%, respectively. Carbonation depth imposes a stronger effect on peak stress than RBA content, whereas strength-growth rate decreases with carbonation progress. Peak strain decreases with increasing carbonation depth but rises with higher RBA replacement ratios, and RBA addition improves concrete ductility. Based on experimental data, a constitutive model for MRBAC is proposed, which simultaneously accounts for the coupled effects of carbonation depth and RBA replacement ratio. This study provides a theoretical basis for the structural design and popularization of MRBAC under carbonation-service environments.

## 1. Introduction

The discharge of construction solid waste has surged in recent years, accounting for 30–40% of municipal solid waste, among which waste concrete and sintered clay bricks constitute the two dominant fractions. The recycling of concrete and brick residues generated from building demolition has thus become a critical research topic in the construction industry. Producing recycled concrete by crushing construction waste into aggregates offers dual benefits: it mitigates environmental pollution from waste stockpiling and curtails excessive exploitation of natural raw materials, alongside cutting carbon emissions from concrete production. Consequently, manufacturing mixed recycled coarse aggregates from waste concrete and waste bricks represents a viable strategy to resolve the disposal dilemma of construction solid waste.

Concrete undergoes carbonation when exposed to atmospheric CO_2_, which lowers the internal alkalinity of concrete and gradually deteriorates the passive film on reinforcing steel. Once the passive film fails to provide protection, water and oxygen penetrate to corrode steel reinforcements. Corrosion-induced volume expansion generates longitudinal cracks along steel bars and spalling of concrete cover, which reduces the cross-sectional area and load-bearing capacity of structural members, ultimately triggering component damage or failure. It is therefore of great theoretical and engineering significance to prepare mixed recycled brick-aggregate concrete (MRBAC) and investigate its carbonation resistance and mechanical performance.

Existing studies [1,2,3,4] have reached a consensus that higher replacement ratios of recycled coarse aggregates (RCAs) lead to inferior carbonation resistance of recycled aggregate concrete (RAC). Huang et al. [5] found that under identical mix proportions, the 28 d carbonation depth of fully recycled aggregate concrete is 11–17% greater than that of normal concrete, and carbonation depth increases nearly linearly as RCA replacement rises from 0% to 100%. Pedro et al. [6] explored the effect of RCA crushing degree on carbonation resistance; their results revealed that concrete manufactured with twice-crushed RCA exhibits slightly lower carbonation depth compared with once-crushed RCA. Xiao et al. [7] evaluated the carbonation performance of RAC incorporating RCA sourced from parent concrete of varying strengths. Deng [8] highlighted that water–cement ratio is a decisive factor governing carbonation depth, with lower water–cement ratios improving carbonation resistance.

Bairagi et al. [9] observed similar stress–strain curve profiles for RAC with different aggregate replacement ratios but did not develop predictive constitutive models. Topçu [10] reported that rising RCA content reduces the elastic modulus of RAC; the descending branches of stress–strain curves differ noticeably across mix designs, and the area enclosed by curves and the horizontal axis diminishes with increased RCA dosage. Tang et al. [11] investigated the combined influence of recycled coarse and fine aggregates on compressive stress–strain responses using Digital Image Correlation (DIC). All specimens exhibited typical shear failure modes, and an empirical stress–strain model was established for fully recycled aggregate concrete. Deng et al. [12] proposed a piecewise cubic polynomial function to describe the ascending and descending branches of uniaxial compressive curves of RAC. Zhao [13] discovered that raising the strength grade of RAC from C20 to C40 steepens the ascending slope, elevates peak stress, and slightly decreases peak strain. Abbara et al. [14] demonstrated that conventional concrete constitutive models fail to accurately capture the stress–strain behavior of mixed recycled aggregate concrete.

Numerous investigations have demonstrated that the physical morphology, porosity and surface texture of waste-derived aggregates strongly govern the fresh-state workability, pore structure and long-term durability of eco-friendly concrete systems. Oliveira et al. [15] investigated the feasibility of utilizing blasted copper slag as artificial fine fractions in cement-based composites, pointing out that waste particles with glassy smooth surfaces can improve mixture workability via ball-bearing effects, while appropriate filler effects contribute to matrix densification; nevertheless, excessive waste dosage may induce degradation of mechanical performance. For construction–demolition waste-originated recycled aggregates, similar mechanisms also apply. Porous brick-containing recycled aggregates introduce additional internal pore channels, which will not only influence mechanical properties but also alter carbon-dioxide diffusion behavior inside concrete. Although extensive research has been conducted on mechanical properties and carbonation resistance of single-type recycled aggregate concrete, few studies focus on MRBAC, and no existing literature has established stress–strain constitutive models for MRBAC after carbonation. To fill the above-mentioned research gap, this study systematically conducts accelerated carbonation tests and uniaxial compression tests on mixed recycled brick-aggregate concrete (MRBAC). Four recycled brick-aggregate replacement ratios (0%, 10%, 20%, 30%) and four accelerated carbonation durations (0 d, 40 d, 80 d, 120 d) are selected as key variables. This work aims to reveal the coupled mechanisms of recycled brick-aggregate content and carbonation depth on carbonation resistance, compressive strength, elastic modulus, and uniaxial compressive stress–strain behavior of MRBAC. On this basis, a phenomenological uniaxial compressive constitutive model considering dual factors is established for carbonated MRBAC. The core new findings indicate that carbonation can effectively compensate part of the mechanical performance loss induced by brick aggregates; carbonation depth exhibits a more dominant influence on peak stress compared with brick-aggregate replacement ratio. This paper can offer fundamental data and theoretical reference for the engineering application of MRBAC made from construction waste.

## 2. Experimental Program

### 2.1. Raw Materials

#### 2.1.1. Cement

P·O 42.5 ordinary Portland cement manufactured by Anhui Conch Group Co., Ltd. (Wuhu, China). was adopted in this test. Its physical, mechanical, and chemical compositions comply with the Chinese national standard GB 175-2023 [16], as summarized in Table 1 and Table 2.

#### 2.1.2. Coarse Aggregates

**Figure 1:**
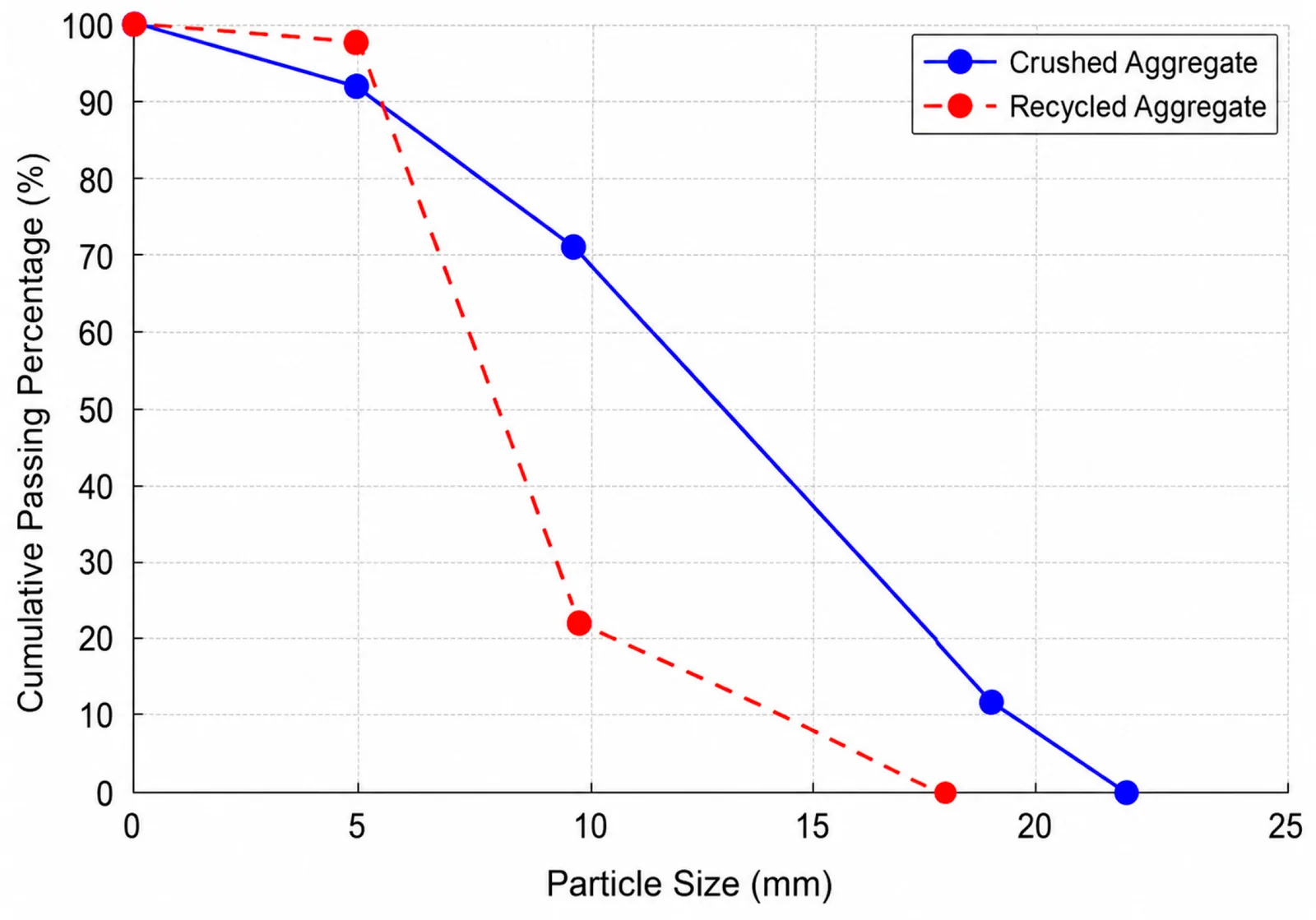
Cumulative sieve residue grading curves of recycled coarse aggregates.

Two types of recycled coarse aggregates were used: recycled concrete aggregate (RCA) and recycled brick aggregate (RBA) from Nanjing Shoujia Renewable Resources Utilization Co., Ltd. (Nanjing, China). Both adopt continuous grading with nominal particle size less than 25 mm; their grading curves are plotted in Figure 1. RCA and RBA were produced via mechanical crushing of waste concrete and sintered clay bricks, respectively.

Fundamental physical properties of RCA and RBA were tested following JGJ 52-2006 [17] and GB/T 25177-2010 [18], with test results listed in Table 3. Considering the high water-absorption characteristic of recycled aggregates, pre-wetting treatment and additional water dosage were adopted during concrete mixing to offset water absorption of RCA and RBA, as described in Section 2.1.4 Mix Proportion. The carbonation degree of the raw recycled aggregates was not measured before specimen casting.

#### 2.1.3. Fine Aggregate

Natural river sand was utilized as fine aggregate, satisfying all specifications in JGJ 52-2006 [17]. The sand exhibits a fineness modulus of 2.60, bulk density of 1666 kg/m^3^, and apparent density of 2581 kg/m^3^.

#### 2.1.4. Mix Proportion

All MRBAC mixtures were designed with a fixed water–cement ratio of 0.52 and target strength grade C25, in accordance with JGJ 55-2011 [19]. Pre-wetting and additional water dosage were adopted to compensate for the high water absorption of recycled aggregates. Four RBA replacement ratios (0%, 10%, 20%, 30%) were designed, denoted as MRBAC0, MRBAC10, MRBAC20, and MRBAC30. Detailed mix proportions are presented in Table 4.

### 2.2. Specimen Fabrication and Curing

A full factorial test matrix was established with 4 RBA replacement ratios × 4 carbonation durations, generating 16 test groups. Two types of specimens were fabricated for each mixture proportion: 100 mm cubic specimens (12 pieces per group) for measuring the carbonation depth and cubic compressive strength of recycled aggregate concrete before and after carbonation; and 100 mm × 100 mm × 300 mm prismatic specimens (6 pieces per group) to obtain the elastic modulus as well as uniaxial compressive stress and strain of recycled concrete. The specimen design scheme is listed in Table 5. Different specimen sizes are adopted to satisfy the Chinese standard GB/T 50081 2019 requirements for different test indices.

The inner surfaces of the mold were coated with lubricant before casting. Raw materials were fully mixed in a horizontal concrete mixer, and fresh concrete was poured into molds followed by vibration table compaction until dense surfaces were achieved. Specimens were demoulded after 24 h of initial setting. Post-demoulding curing lasted 7 d under ambient conditions of 20 ± 2 °C and relative humidity 60% ± 5% with regular water spraying, after which specimens were cured under natural outdoor environments for an additional 21 d to reach a total curing age of 28 d prior to accelerated carbonation exposure.

### 2.3. Test Methods

#### 2.3.1. Accelerated Carbonation Test

After 28 d of standard curing, cubic and prismatic specimens were placed in an HTX-1600G automatic concrete carbonation chamber from Suzhou Donghua Testing Instrument Co., Ltd. (Suzhou, China). Accelerated carbonation tests strictly followed GB/T 50082-2024 [20]. At carbonation ages of 3 d, 7 d, 14 d, 40 d, 80 d, and 120 d, selected specimens were split to measure carbonation depth.

Split cross-sections were cleaned and sprayed with 1% phenolphthalein alcohol indicator solution. After a 30 s reaction period, carbonation depth was measured every 10 mm along specimen edges using a steel ruler, with at least five measuring points per cross-section (precision: 0.5 mm). The average carbonation depth *d*_t_ at carbonation age *t* was calculated via Equation (1):

(1) $$d_{t}=\frac{1}{n}\sum_{i=1}^{n}d_{i}$$

where *d*_t_ = average carbonation depth after t days of carbonation (mm); *d_i_* = carbonation depth measured at point *i* (mm); *n* = total number of measuring points.

#### 2.3.2. Cubic Compressive Strength Test

After reaching target carbonation ages, cubic compressive strength tests were conducted on 100 mm cubic specimens per GB/T 50081-2019 [21]. A size conversion coefficient of 0.95 was applied to convert measured strength to standard cube strength, with a constant loading rate of 0.4 MPa/s–0.6 MPa/s. Compressive strength was calculated by Equation (2):

(2) $$f_{\mathrm{cu}}=0.95\frac{F}{A}$$

where *f*_cu_ = cubic compressive strength (MPa); *F* = ultimate failure load (N); *A* = bearing cross-sectional area (mm^2^).

#### 2.3.3. Uniaxial Compression Test for Prismatic Specimens

**Figure 2:**
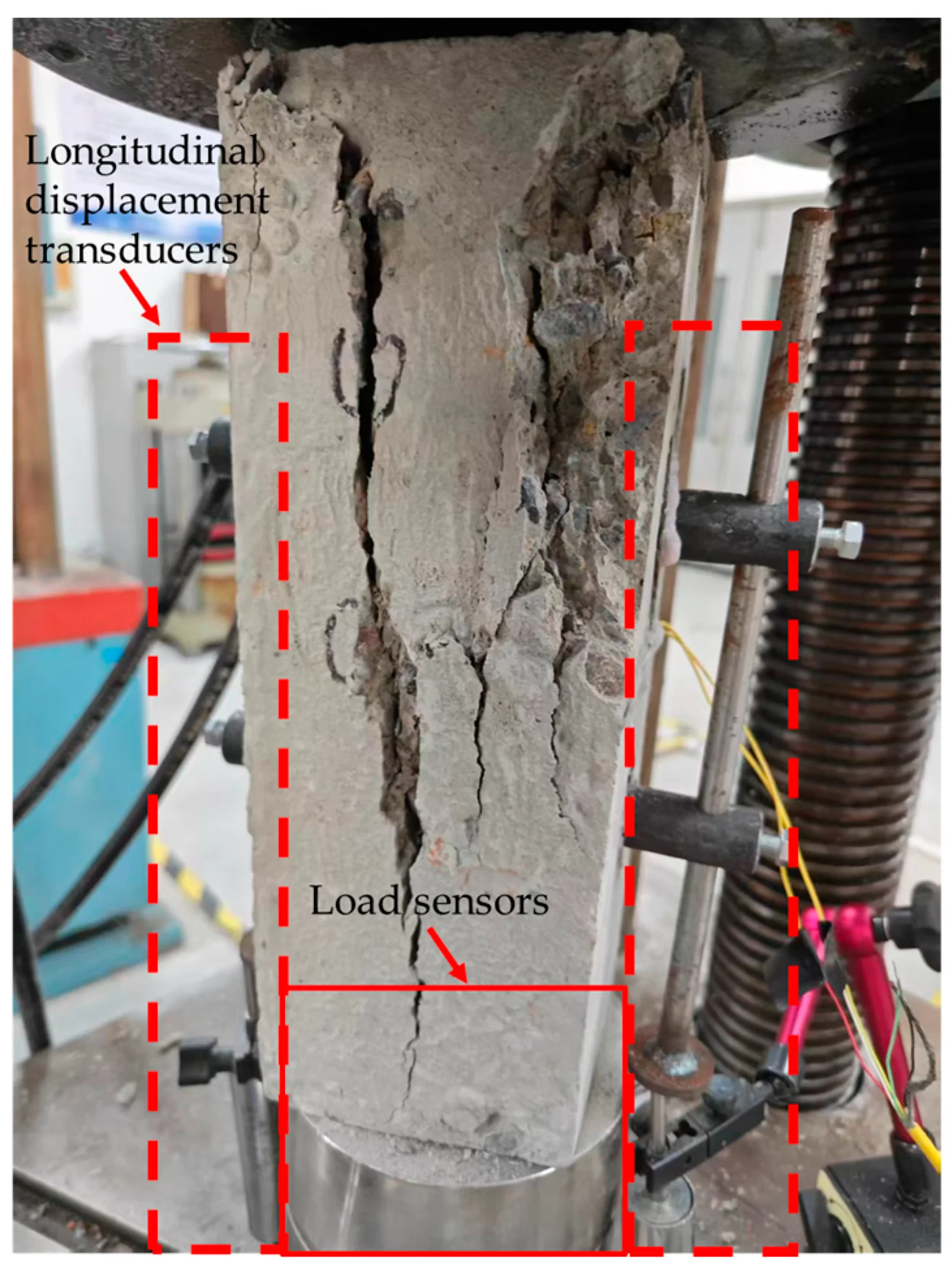
Layout of displacement measurement devices on prismatic specimens.

Uniaxial monotonic compression tests on 100 mm × 100 mm × 300 mm prisms complied with GB/T 50081-2019 [21]. The elastic modulus and Poisson’s ratio were measured together with the full-range uniaxial compressive stress–strain curves. The test setup consisted of a hydraulic loading frame, load sensors, longitudinal displacement transducers, strain gauges (Chengdu Siweide Technology Co., Ltd., Chengdu, China), and a high-speed dynamic data acquisition system (Jiangsu Donghua Testing Technology Co., Ltd., Taizhou, China), as illustrated in Figure 2.

## 3. Experimental Results and Discussion

### 3.1. Carbonation Depth of MRBAC

**Figure 3:**
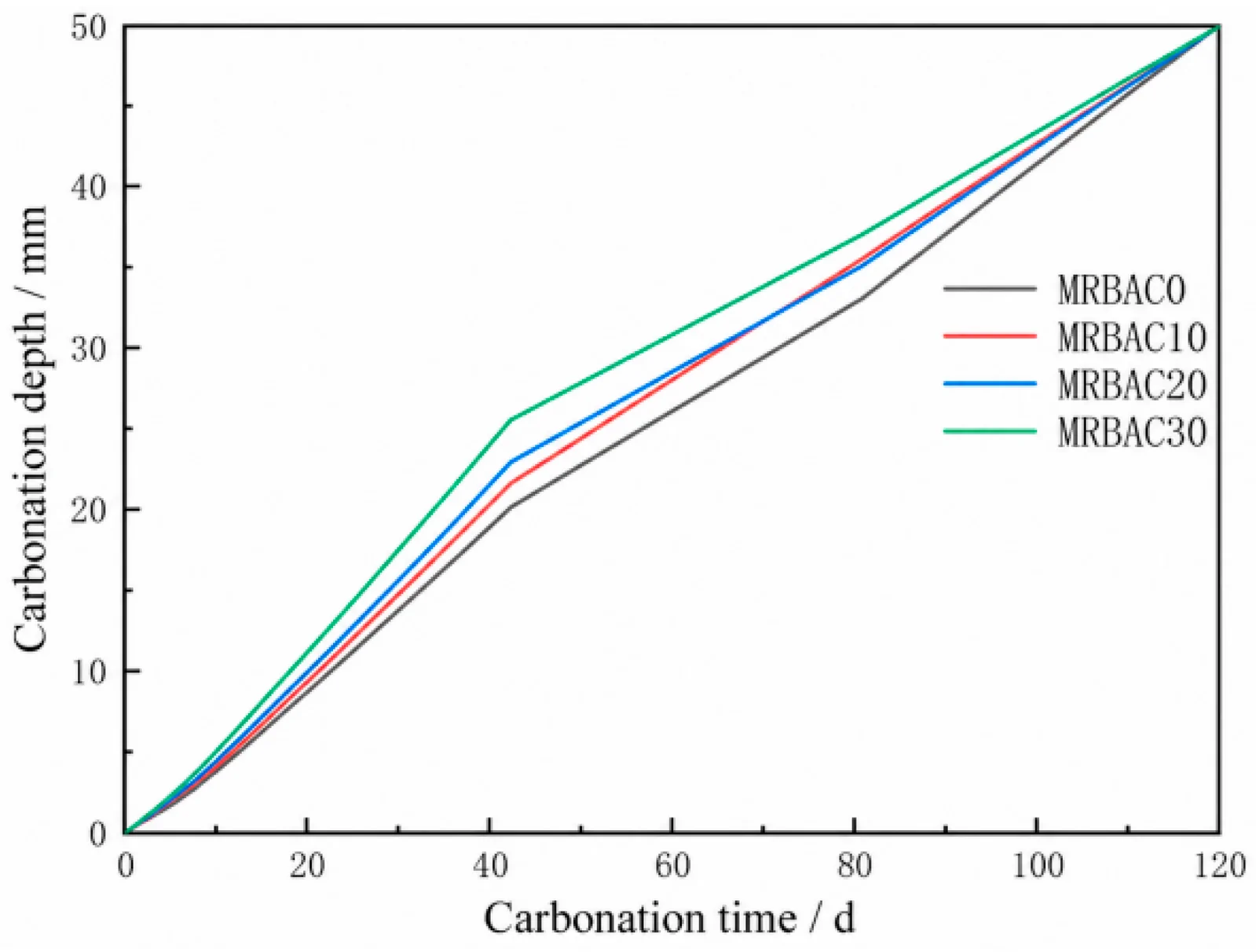
Variation of carbonation depth with carbonation time for specimens.

Figure 3 presents the evolution of average carbonation depth against carbonation duration for all MRBAC mixtures. The carbonation depth of MRBAC is listed in Table 6. For all fixed RBA replacement ratios, carbonation depth increases monotonically with prolonged carbonation exposure. It can be observed from the test results that for MRBAC with a fixed recycled aggregate replacement ratio, the carbonation depth increases monotonically as carbonation duration is extended. For MRBAC0 specimens subjected to identical curing ages, their carbonation depth rises from 20.46 mm to 33.20 mm after 40 d and 80 d of accelerated carbonation, representing an increment of 62.2%. For MRBAC10 specimens with the same curing age, the carbonation depth increases from 21.88 mm to 35.66 mm after 40 d and 80 d of carbonation, with a growth rate of 63.1%. For MRBAC20 specimens of equal age, the carbonation depth grows from 23.14 mm to 35.18 mm following 40 d and 80 d carbonation, an increase of 74.7%. For MRBAC30 specimens at the same curing age, the carbonation depth expands from 23.65 mm to 37.04 mm after 40 d and 80 d carbonation, corresponding to a 62.2% rise.

**Figure 4:**
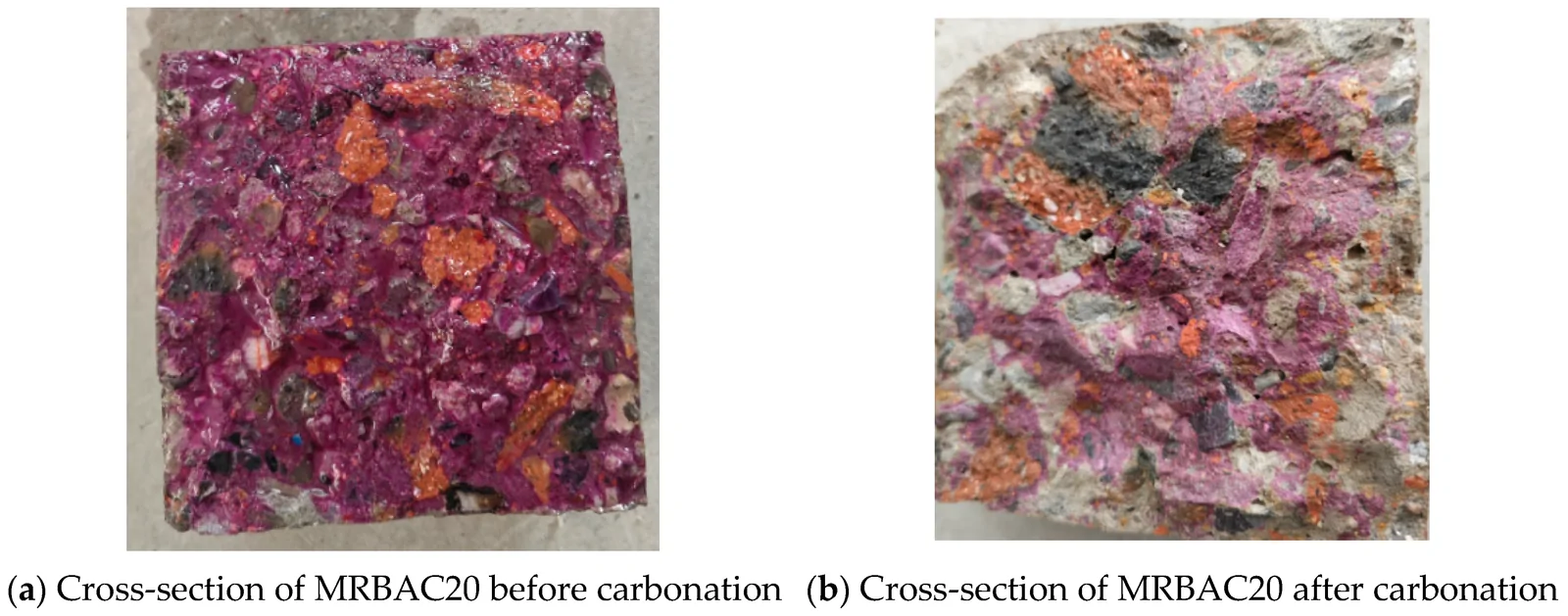
Cross-sectional phenolphthalein staining of MRBAC20 specimens: (**a**) Cross-section of MRBAC20 before carbonation; (**b**) Cross-section of MRBAC20 after carbonation.

As shown in Figure 4, after 40 days of carbonation, the uncarbonated regions are primarily composed of recycled concrete aggregates. In contrast, the hydration products inside brick aggregates and the surrounding mortar layers have been neutralized by carbonation in advance. Accordingly, the RBA content exerts a significant influence on carbonation depth. After 80 days of carbonation, the carbonation depth of MRBAC30 is approximately 13% higher than that of MRBAC0.

Bricks are manufactured via high-temperature sintering, so they generally possess higher porosity than natural crushed stone, accompanied by looser interfacial transition zones (ITZs) between bricks and cement paste. As the replacement ratio rises, the aggregate composition becomes more complex, which expands and interconnects internal pores. Carbon dioxide can rapidly diffuse inward through these interconnected voids and accelerate the carbonation process. The rough, porous surfaces of brick aggregates prevent complete filling by mortar, forming looser ITZs between RBA and both new and old mortar. A higher RBA replacement ratio corresponds to a larger volume fraction of such weak ITZs. Since ITZs act as the primary pathways for CO_2_ penetration, the penetration resistance of CO_2_ is further reduced, resulting in greater carbonation depth with increasing replacement ratio.

The progression of carbonation reactions relies on the penetration efficiency of CO_2_, and higher RBA content provides additional transport channels for CO_2_, thus accelerating inward carbonation. Although calcium carbonate (CaCO_3_) generated during carbonation can partially fill mortar pores, it cannot effectively block the interconnected intrinsic pores of brick aggregates or their fragile ITZs. For this reason, the impact of mixed recycled aggregates on carbonation depth differs from that of single recycled concrete aggregates or natural coarse aggregates.

The RBA adopted in this test features smaller particle sizes than RCAs. Despite the relatively low concentration of carbonatable substances within individual brick particles, their small grain size yields a larger specific surface area. This increases the total volume of aged mortar attached to aggregate surfaces, and more carbonatable substances infiltrate into RBA during cement hydration. Consequently, specimens with higher RBA content exhibit deeper carbonation.

The faster carbonation rate observed in specimens with higher RBA content can be explained from microstructural and chemical perspectives. The porous nature of recycled brick aggregates provides extra transport pathways for CO_2_ ingress, and the loose interfacial transition zones (ITZs) between brick aggregates and cement mortar serve as preferential diffusion channels for carbon-dioxide gas [7]. During carbonation, calcium hydroxide in cement paste reacts with CO_2_ to generate calcium carbonate precipitates, which can partially fill internal pores. Nevertheless, such filling effect cannot completely block the interconnected inherent pores inside brick aggregates, so higher brick-aggregate dosage accelerates the carbonation front advancement. The above trend is consistent with previous findings reported by Huang et al. [5], who stated that increasing recycled aggregate content aggravates the carbonation susceptibility of recycled aggregate concrete. It should be noted that the pore characteristics of raw recycled aggregates vary from source to source; hence, quantitative carbonation results may differ among different research programs.

### 3.2. Cubic Compressive Strength of Carbonated MRBAC

**Figure 5:**
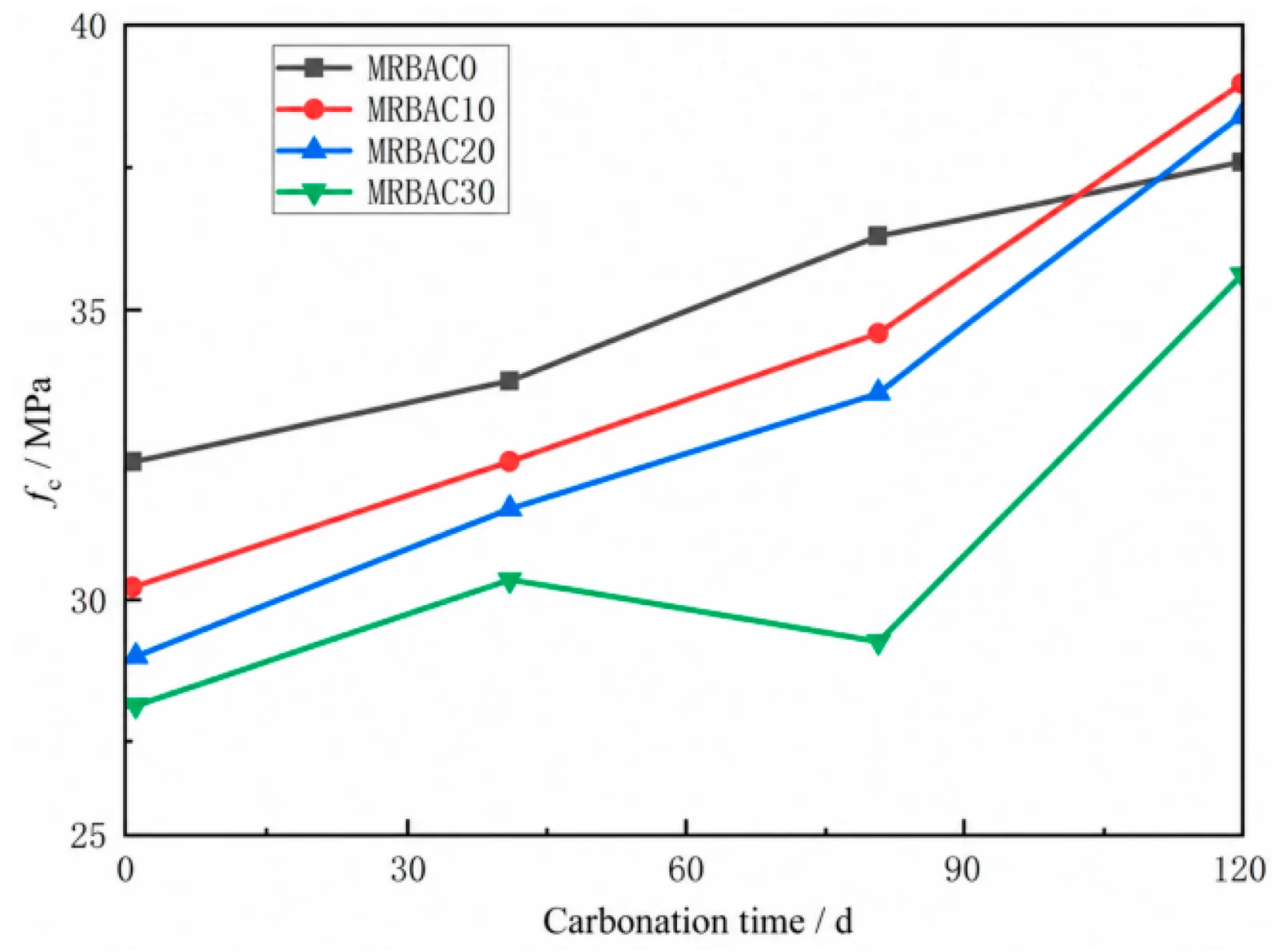
Cubic compressive strength *f*_cu_ of MRBAC under different carbonation durations.

Figure 5 plots the cubic compressive strength development of MRBAC over carbonation time. It can be seen from the test results that the cubic compressive strength *f*_cu_ of MRBAC specimens prepared with various mix proportions rises to different extents as carbonation depth increases after accelerated carbonation tests. After complete 120 d carbonation: the *f*_cu_ of MRBAC0 rises by 16.07%, MRBAC10 by 28.75%, MRBAC20 by 32.13%, and MRBAC30 by 28.24%.

As illustrated in Figure 5 and Table 7, the cubic compressive strength of MRBAC generally declines with the increase in recycled brick-aggregate (RBA) replacement ratio, yet grows with prolonged carbonation duration. The overall strength gain of carbonated MRBAC mainly stems from pore filling of fresh mortar by carbonation products and the densification of interfacial transition zones (ITZs). Nevertheless, brick aggregates possess low intrinsic strength and abundant internal defects; thus, higher RBA replacement ratios lead to smaller strength increments after carbonation, as well as lower ultimate compressive strength under full carbonation.

Figure 5 also reveals obvious discrepancies in cubic compressive strength among MRBAC groups with different RBA replacement ratios at low carbonation depths. The cubic compressive strength of MRBAC0 is 14.7% higher than that of MRBAC30. It can therefore be concluded that the deteriorating effect induced by brick aggregates dominates at low carbonation degrees. Under compression, inherent defects inside brick aggregates (pores, attached aged mortar, microcracks) rapidly expand and interconnect. At the early carbonation stage, only a small amount of carbonation products are generated, which fail to fill internal voids sufficiently; accordingly, carbonation exerts limited reinforcing effects on cubic compressive strength.

When carbonation proceeds to the intermediate stage, its enhancement effect on MRBAC compressive strength becomes prominent. Continuous precipitation of carbonation products fills pores within fresh mortar and ITZs, improving matrix compactness and further elevating strength. The reinforcing effect remains stable for mixtures with low RBA replacement ratios, whereas it is restrained for high-RBA specimens due to abundant internal aggregate defects.

At the late carbonation stage, the influence of carbonation depth becomes more pronounced. All specimens achieve substantial compressive strength growth upon full carbonation, especially those with high RBA contents. For instance, MRBAC20 gains a 14.4% increase in cubic compressive strength over the final 40 d of carbonation, compared with merely 3.6% for MRBAC0 under identical carbonation conditions. This indicates that carbonation delivers a more significant strengthening effect on MRBAC containing more inherent defects. Although the ranking of MRBAC cubic compressive strength remains unchanged after full carbonation, the strength gaps between different groups are narrowed, proving that carbonation partially offsets the adverse mechanical impacts brought by brick aggregate incorporation.

The RBA replacement ratio primarily governs the initial strength and strength ceiling of MRBAC; higher replacement ratios correspond to more internal defects and lower compressive strength. Carbonation depth mainly controls the growth trend and magnitude of concrete strength: carbonation products fill pores to raise compressive strength, with the most remarkable reinforcing performance observed for defect-rich MRBAC at the late carbonation stage. Therefore, both long-term carbonation effects and aggregate replacement ratios should be comprehensively considered in the design of MRBAC structural members. The carbon-induced strength improvement remains stable under low RBA replacement ratios. Despite considerable strength growth observed for high-RBA mixtures, the absolute strength increment is limited, which necessitates a cap on RBA dosage during mix proportion design.

The strength-enhancement effect induced by accelerated carbonation originates from chemical mineral-phase transformation at the microscale. Precipitated calcium carbonate fills capillary pores and microcracks within the cement matrix and ITZs, improving matrix compactness [2]. For MRBAC containing high-content brick aggregates, more initial micro-defects exist inside brick particles and their surrounding interfaces. Carbonation products can fill these weak zones and partially compensate the strength loss caused by porous brick aggregates, which explains why high-RBA groups obtain relatively large strength increments after long-term carbonation. This observation supports the conclusion from Xiao et al. [7] that carbonation exerts more prominent modification for recycled concrete with more initial inner defects. However, the absolute strength of high-RBA specimens still remains lower than that of groups without brick aggregates, because the intrinsically low strength of brick particles cannot be eliminated merely by carbonation reactions.

### 3.3. Elastic Modulus of Carbonated MRBAC

The elastic modulus is calculated following GB/T 50081 2019 [21], using the load corresponding to 1/3 of axial compressive strength, as defined in Equation (3).

(3) $$E_{c}=\frac{F_{a}-F_{0}}{A}\times \frac{L}{\Delta n}$$

where *E*_c_ = elastic modulus (MPa); *F*_a_ = load corresponding to 1/3 axial compressive strength (N); *F*_0_ = initial preloading load (N); *A* = cross-sectional bearing area (mm^2^); *L* = gauge length for displacement measurement (180 mm in this test); and Δ*n* = average longitudinal deformation recorded by two transducers between *F*_0_ and *F*_a_ (mm).

**Figure 6:**
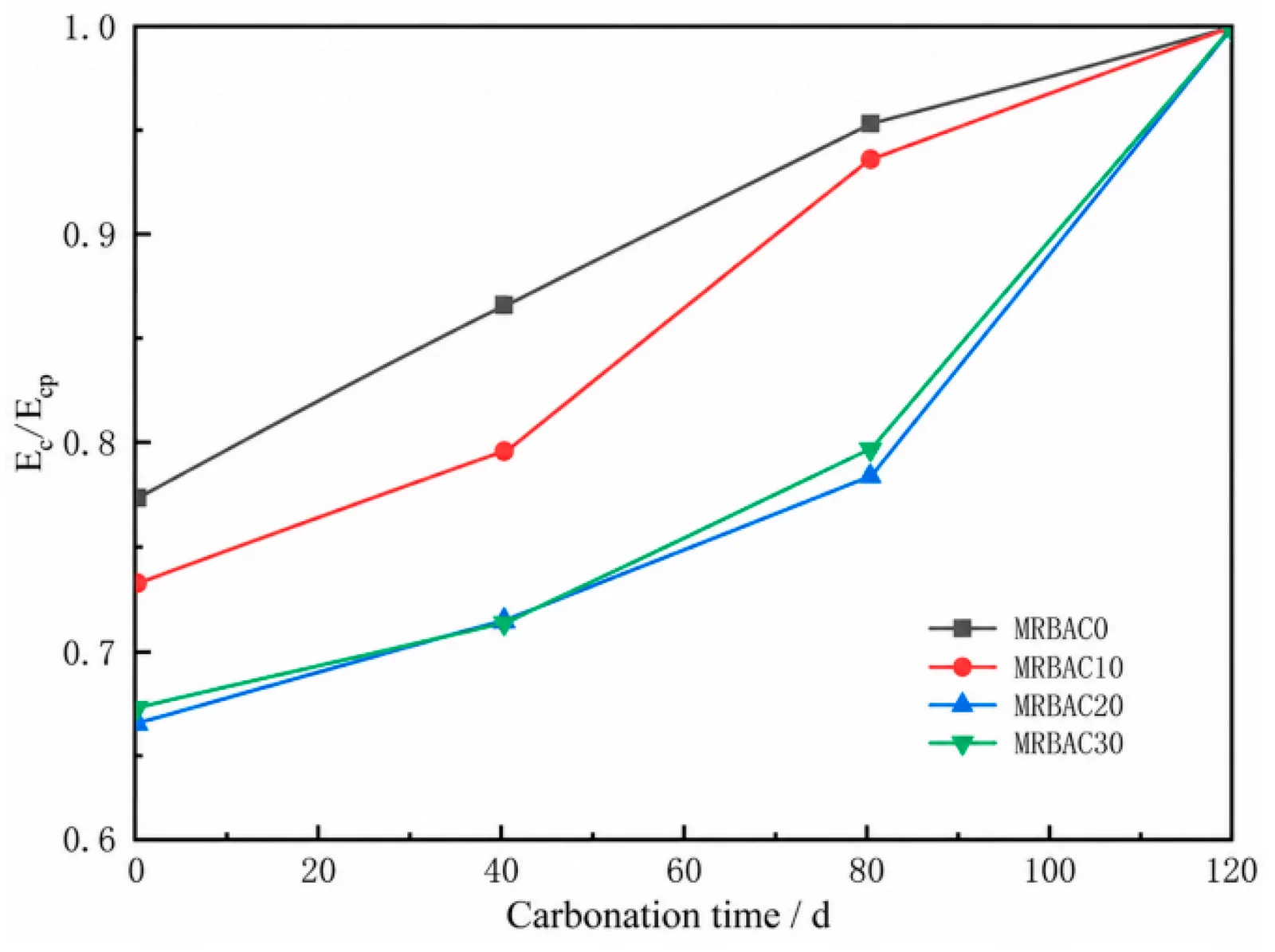
Variation in normalized elastic modulus with carbonation time.

The normalized elastic modulus *E*_c_/*E*_cp_ of carbonated MRBAC is presented in Figure 6 and Table 8. The normalized elastic modulus *E*_c_/*E*_cp_ is defined as the ratio between the measured elastic modulus *E*_c_ at a given carbonation age and the elastic modulus *E*_cp_ of the fully carbonated specimen. It can be observed from Figure 6 that the elastic modulus of MRBAC0 increases by 28.3% during the carbonation process from the uncarbonated state to full carbonation. Furthermore, the increment of elastic modulus in the early carbonation stage is larger than that in the later stage: the elastic modulus rises by 2.9 GPa within the first 40 days of carbonation, whereas only a 1.5 GPa increment is achieved in the final 40 days. After full carbonation, the elastic modulus of MRBAC10 increases by 36.0%; MRBAC20 shows a 50.1% increase, and MRBAC30 exhibits a 48.0% increase. Regardless of the replacement ratio of recycled brick aggregate (RBA), the elastic modulus increases monotonically with prolonged carbonation time.

At identical carbonation ages, the elastic modulus decreases monotonically with the increase in RBA content, with the reduction ranging from 31.8% to 41.4%. This phenomenon arises because the elastic modulus of brick aggregates is considerably lower than that of natural aggregates (the elastic modulus of brick is approximately 20 GPa–30 GPa). As the proportion of brick aggregates increases, the volume fraction of coarse aggregates with low elastic modulus in concrete rises, imposing a stronger degradation effect on the overall stiffness and thus resulting in a continuous decline in elastic modulus.

When both RBA content and carbonation time vary simultaneously, carbonation leads to an increased elastic modulus for all mixtures irrespective of RBA replacement level, and the growth margin slightly rises with increasing brick-aggregate content. This is attributed to the weaker interfacial transition zone (ITZ) of RBA, which is more susceptible to carbonation reactions. MRBAC with high RBA content possesses the lowest elastic modulus prior to carbonation, yet the absolute increment in elastic modulus after carbonation is comparable to groups incorporating low brick-aggregate content. This indicates that although brick aggregates reduce the initial stiffness, their high porosity enables calcium carbonate (CaCO_3_) generated by carbonation to penetrate pores more effectively and exert a filling effect, strengthening the overall stiffness of specimens. Consequently, the improved elastic modulus becomes close to that of MRBAC0.

The evolution trend of elastic modulus for MRBAC conforms to classical theories: slight growth in elastic modulus generally accompanies strength gain at the early carbonation stage, followed by a slow reduction in elastic modulus induced by strength degradation in the later phase. Nevertheless, the elastic modulus of MRBAC0 continuously rises after carbonation in this study. A plausible explanation is that recycled aggregates exhibit high water absorption; water expulsion during carbonation further promotes pore densification, offsetting the adverse effect of late-stage strength loss. For concrete incorporating brick aggregates, the low elastic modulus and microporous structure of brick aggregates render them more sensitive to carbonation. Carbonation products not only fill internal pores but also enhance the compactness of the brick–cement ITZ. Therefore, despite the lowered initial stiffness, brick aggregates amplify the stiffness enhancement effect induced by carbonation.

Existing studies generally acknowledge that the elastic modulus of recycled aggregate concrete gradually decreases with the rising replacement ratio of recycled coarse aggregates [22,23]. This is mainly attributed to microcracks generated during the production of recycled aggregates and residual old mortar attached to aggregate surfaces, which reduce the stiffness of aggregates themselves and further impair the overall elastic modulus of concrete. Research findings from most scholars demonstrate that the elastic modulus is positively correlated with the cubic compressive strength in empirical formulas. In this work, a correction coefficient related to the RBA replacement ratio is introduced into the formula proposed by Hao and Ren [23] to predict the elastic modulus of MRBAC, as expressed in Equation (4).

(4) $$E_{c}=k_{b}\frac{10^{5}}{a+\frac{b}{f_{\mathrm{cu}}}}$$

where *k*_b_ is the influence coefficient of brick aggregate; a and b are correction coefficients; *f*_cu_ is the cubic compressive strength.

Based on the experimental data measured in this study, regression analysis on the relationship between cubic compressive strength and elastic modulus of MRBAC is carried out using Equation (4), yielding:

(5) $$\begin{array}{l} E_{c}=\frac{10^{5}}{-0.05+\frac{2.69}{f_{\mathrm{cu}}}}\\ R^{2}=0.925 \end{array}$$

**Figure 7:**
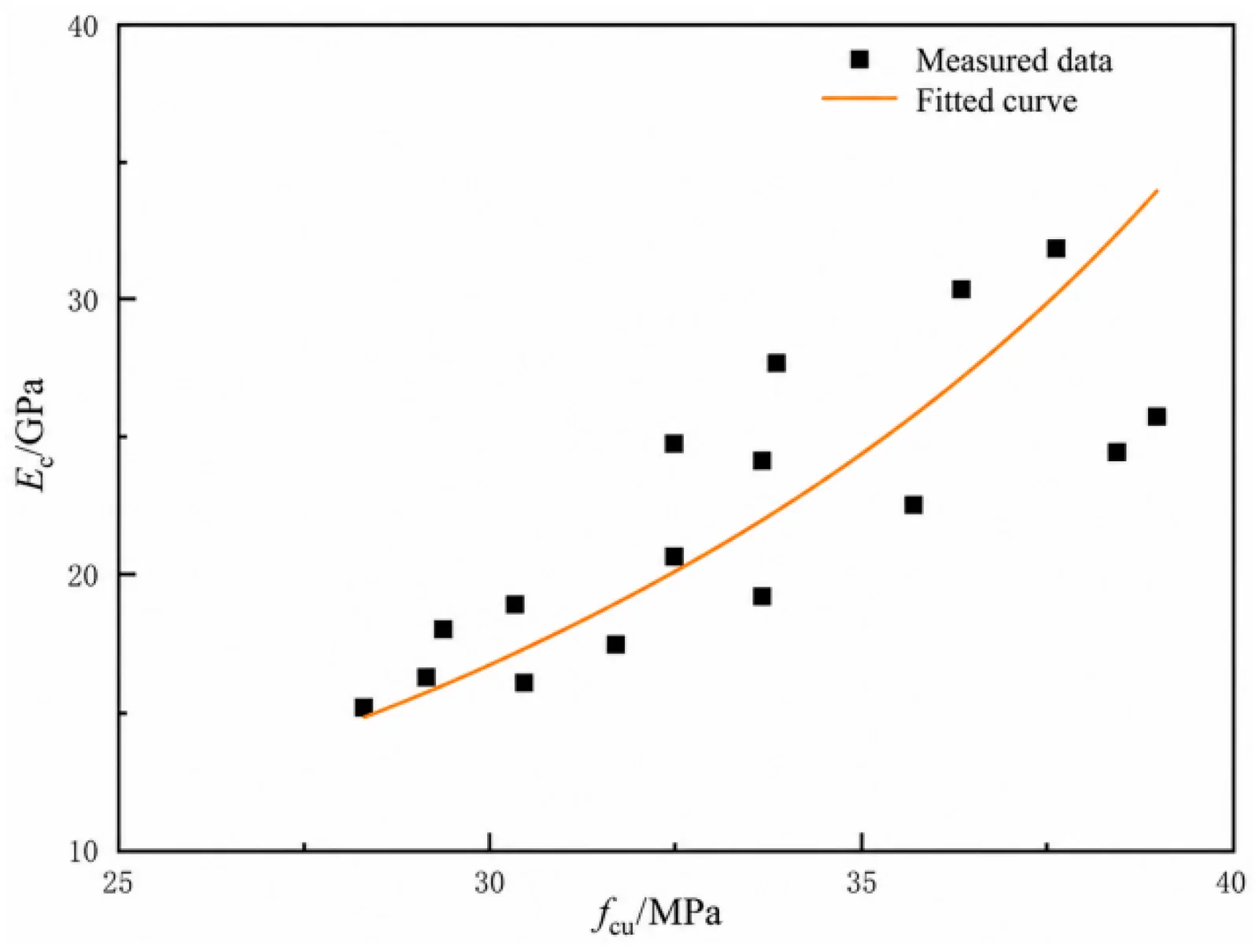
The comparison between fitting results and measured data.

The comparison between fitting results and measured data is shown in Figure 7. It can be seen from Figure 7 that the proposed model provides satisfactory predictions for the elastic modulus of MRBAC.

The reduction in elastic modulus with increasing RBA replacement ratio is dominated by composite material micromechanics. Recycled brick aggregates possess inherently lower elastic modulus compared with natural stone and recycled concrete aggregates, and their addition reduces the overall stiffness of concrete composite [22]. Meanwhile, porous brick-mortar ITZs represent weak stiffness-controlling regions. Carbonation reactions densify these loose interfacial zones via carbonate deposition, leading to continuous modulus growth even for high-RBA specimens. The modulus-evolution tendency in this test agrees with Adessina et al. [22], who reported that recycled aggregate concrete gains stiffness improvement after carbonation due to microstructural densification. It is worth noting that modulus improvement brought by carbonation cannot fully offset the stiffness degradation originating from brick-aggregate incorporation.

### 3.4. Uniaxial Compressive Test on Prismatic MRBAC Specimens After Carbonation

#### 3.4.1. Failure Process of Specimens

**Figure 8:**
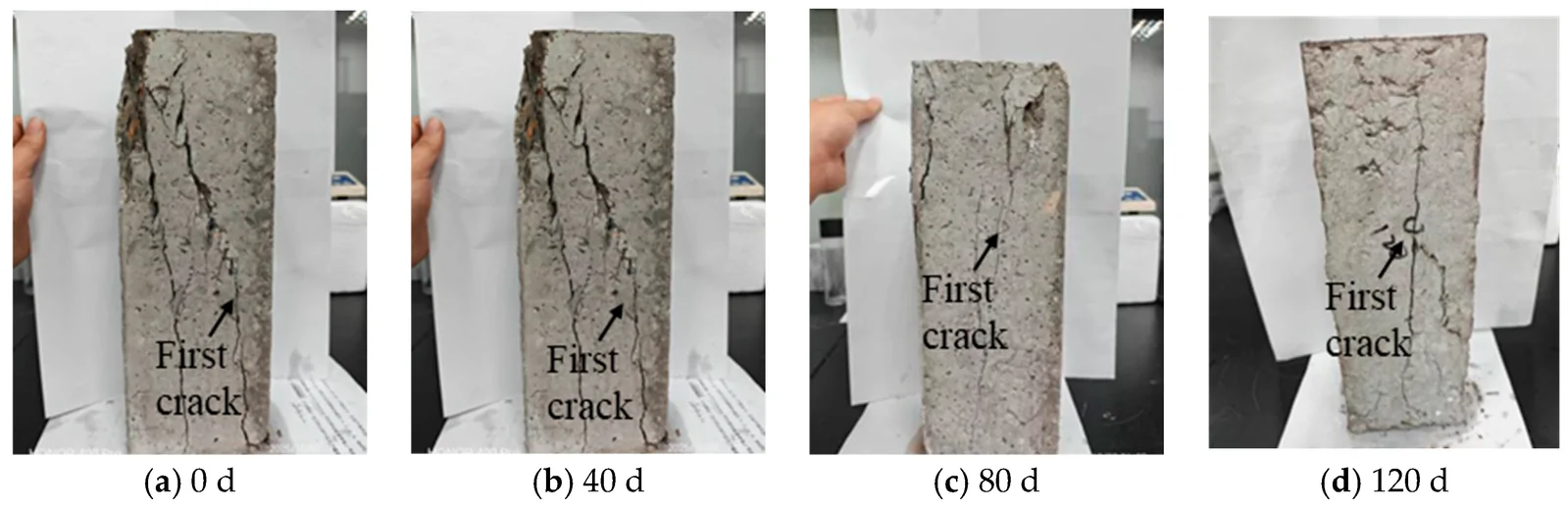
Failure pattern of MRBAC0: (**a**) 0 d; (**b**) 40 d; (**c**) 80 d; (**d**) 120 d.

**Figure 9:**
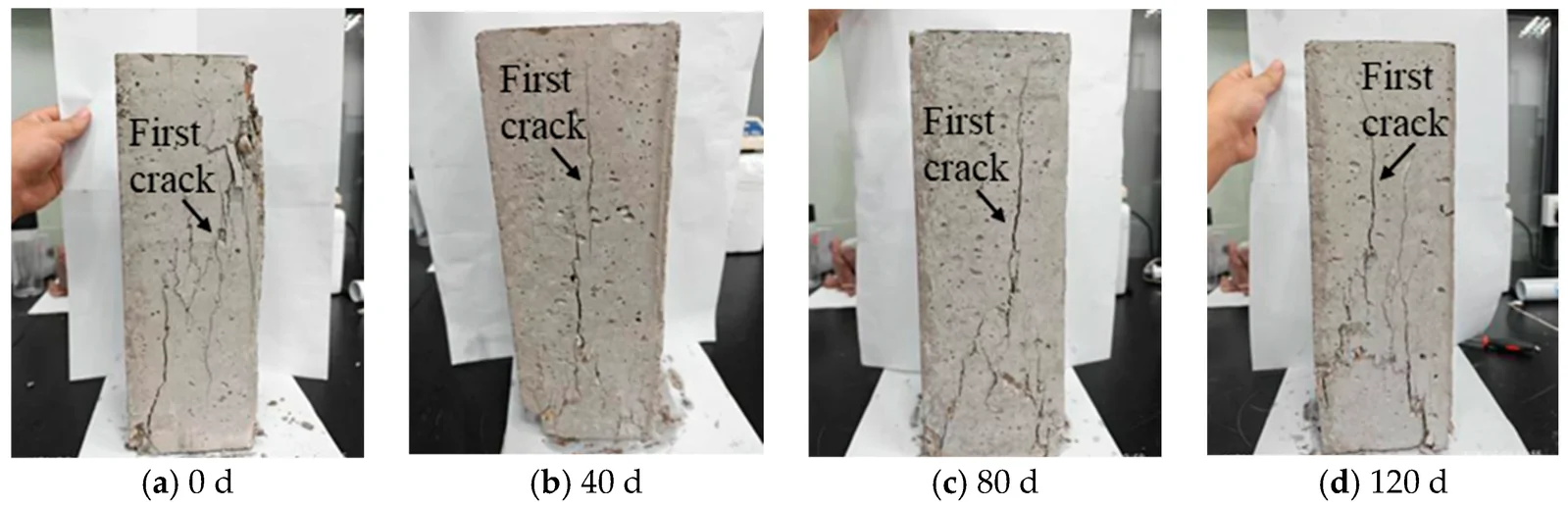
Failure pattern of MRBAC10: (**a**) 0 d; (**b**) 40 d; (**c**) 80 d; (**d**) 120 d.

**Figure 10:**
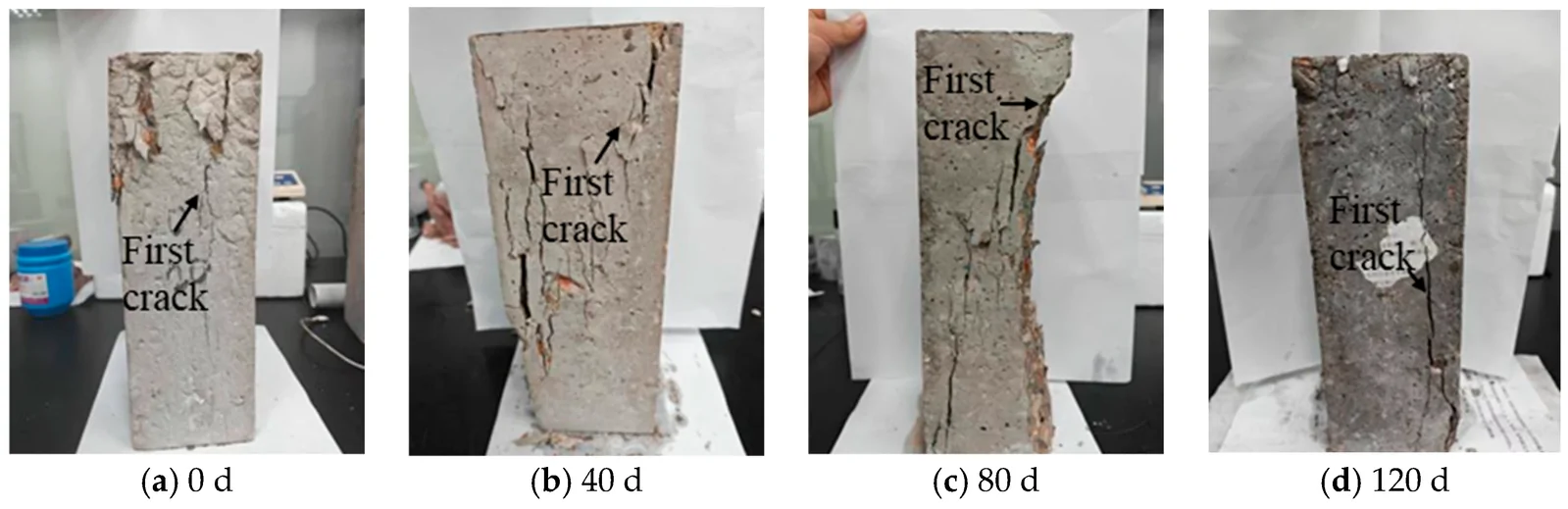
Failure pattern of MRBAC20: (**a**) 0 d; (**b**) 40 d; (**c**) 80 d; (**d**) 120 d.

**Figure 11:**
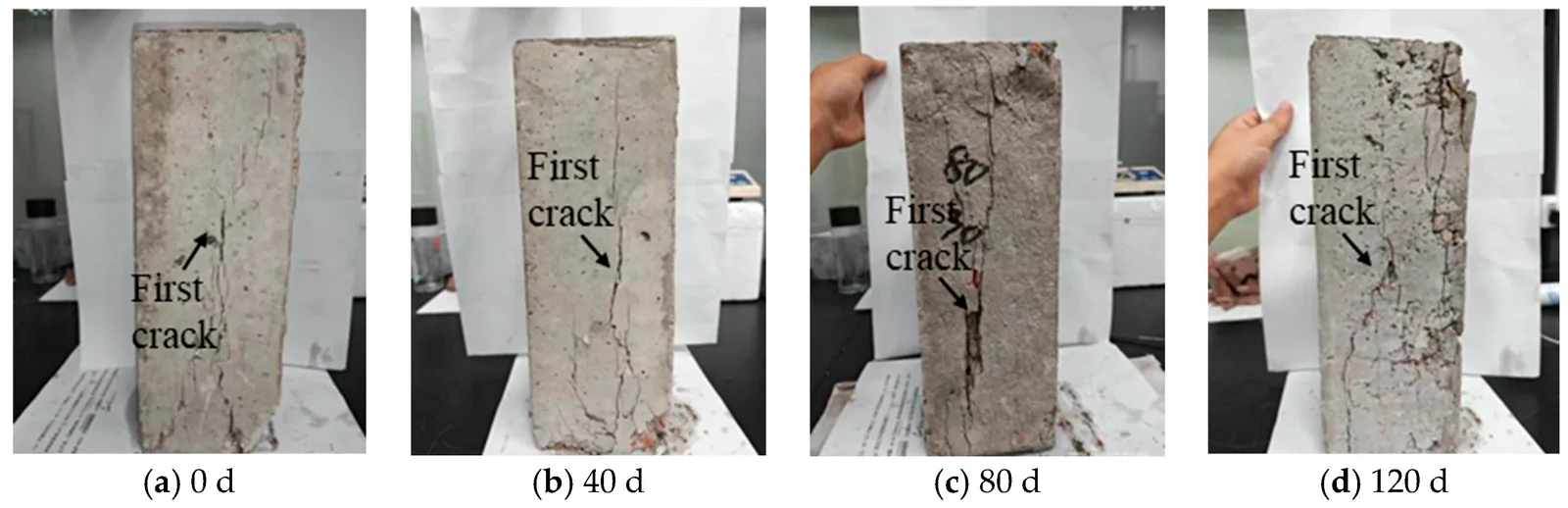
Failure pattern of MRBAC30: (**a**) 0 d; (**b**) 40 d; (**c**) 80 d; (**d**) 120 d.

Carbonated MRBAC exhibits a similar failure process to normal aggregate concrete (NAC). From the onset of loading until the applied load reaches 0.3 times the peak stress of the specimen, the specimen remains in an elastic state without obvious surface changes. The load readings show a good linear relationship with the displacement readings acquired by sensors. Slight sounds can be heard as the specimen is compressed. When the axial load reaches 0.8 times the peak stress, tiny, discontinuous short cracks initiate on the side surfaces of the specimen. This stage corresponds to stable crack propagation. Stress increases rapidly in this process, and the load–displacement relationship begins to exhibit nonlinear characteristics. As the axial load approaches the peak stress, vertical cracks parallel to the loading direction emerge on the specimen surface. Initial cracks initiate near the mid-height of specimens, and no obvious torsional deformation is observed during compression tests. The initial fine short cracks gradually extend and widen, interconnecting with other cracks to form several visible longitudinal or slightly inclined cracks. At this critical stage, the growth rate of the load slows down remarkably, indicating the precursor of failure. After the axial load reaches the peak stress, surface cracks connect and form a major diagonal through crack roughly along the surface diagonal of the specimen. Failure occurs, accompanied by spalling of the concrete surface layer. Small pieces of mortar or interfacial composites of old and new mortar detach first from the crack edges. Compared with ordinary concrete, the final failure morphology is more fragmented and loose, with missing corners. The failure patterns of carbonated specimens are presented in Figure 8, Figure 9, Figure 10 and Figure 11.

The failure modes of MRBAC are closely related to its multi-phase micro-defect distribution. Cracks preferentially propagate along weak brick-mortar interfacial transition zones under compressive loading. After carbonation, the densified ITZs change crack propagation paths and increase material brittleness, which corresponds to the more sudden failure phenomenon observed in carbonated specimens. Similar failure-mode characteristics for recycled aggregate concrete were described by Tang et al. [11].

#### 3.4.2. Measured Stress–Strain Curves

A dynamic data acquisition system was adopted to record the axial compressive load F_V_ and longitudinal compressive deformations R_V1_ and R_V2_ within the gauge length of 180 mm during the uniaxial compressive test of specimens.

The compressive stress of specimens is calculated by Equation (6):

(6) $$\sigma =\frac{P_{v}}{A}=\frac{P_{v}}{100\times 100}=10^{-4}P_{v}$$

where *σ* = compressive stress of the specimen; *P*_v_ = vertical load measured by the testing machine; *A* = compressive cross-sectional area of the specimen.

The compressive strain *ε* of specimens is determined by Equation (7):

(7) $$\varepsilon =\frac{R_{v1}+R_{v2}}{2R_{v}}=\frac{R_{v1}+R_{v2}}{360}$$

where *ε* = compressive strain of the specimen; *R*_v1_ and *R*_v1_ = longitudinal displacements measured by two displacement sensors; the gauge length *R*_v_ of the displacement sensors is 180 mm.

**Figure 12:**
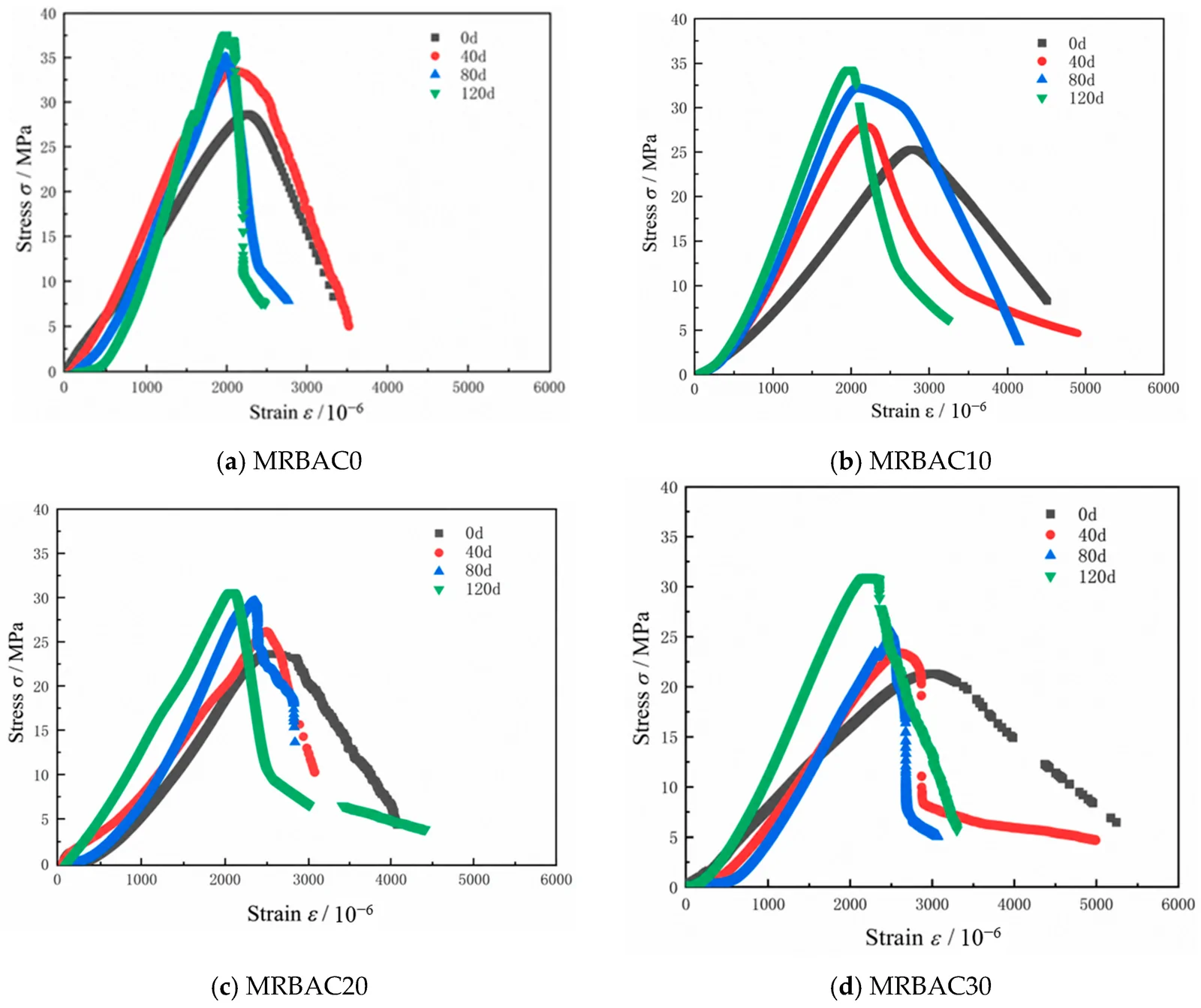
Stress–strain curves of MRBAC with different replacement ratios of recycled brick aggregate: (**a**) MRBAC0; (**b**) MRBAC10; (**c**) MRBAC20; (**d**) MRBAC30.

Based on the measured stress and strain data, the uniaxial compressive stress–strain curves of MRBAC specimens are calculated via Equations (6) and (7). Using the obtained results, stress–strain curves of MRBAC under different recycled brick-aggregate (RBA) replacement ratios and various carbonation durations are plotted in Origin 2024, as illustrated in Figure 12. The concavity observed at the initial loading stage is mainly caused by the compaction of gaps between specimen surface, loading platen and displacement transducers at the beginning of loading.

As shown in Figure 12, the peak stress point on the stress–strain curve shifts upward for all MRBAC mixtures with longer carbonation duration, indicating that carbonation improves the stiffness of MRBAC. Meanwhile, the ascending branch of the curve shifts leftward overall, accompanied by a reduction in peak strain corresponding to peak stress, which demonstrates weakened plastic characteristics of specimens.

At identical carbonation duration, the initial slope of the stress–strain curve of MRBAC gradually decreases with the increase in RBA content. Within the elastic stage, the slope rises as carbonation proceeds, revealing enhanced initial stiffness of carbonated recycled concrete. In the elastoplastic ascending stage, the evolution trend of stress–strain curves of MRBAC generally resembles that of ordinary concrete. The curve deviates from linearity and rises convexly until reaching peak stress, during which internal microcracks develop stably. For longer carbonation time, the stress–strain curve remains closer to a straight line before peak stress. This indicates that carbonation delays the initiation and unstable propagation of internal microcracks. The densification induced by carbonation strengthens the interfacial transition zone (ITZ) of the matrix and raises the stress required for microcrack initiation, enabling the material to sustain higher loads without significant damage. Correspondingly, higher brick-aggregate content leads to earlier deviation from linearity and more pronounced nonlinearity. This implies that cracks initiate and propagate stably at an early loading stage. The mixed coarse aggregates generate complex interfacial transition zones not only between new-old cement mortar and aggregates but also between recycled concrete aggregates (RCAs) and RBA. These interfaces serve as preferential crack propagation paths under relatively low stress, resulting in premature plastic deformation.

The curve after the peak stress directly reflects the deformation characteristics of specimens. The steepness of the descending branch characterizes the brittleness of materials. It can be observed from Figure 12 that fully carbonated specimens exhibit steeper descending branches compared with uncarbonated specimens. This reveals that carbonation reduces the ductility of recycled concrete, leading to lower residual bearing capacity and a shorter post-peak bearing phase after failure. By contrast, a higher content of brick aggregates yields a gentler descending branch, demonstrating that brick aggregates improve the stress transfer capacity of MRBAC. Owing to the porosity and low bonding strength of brick aggregates, more energy can be absorbed and dissipated during crack propagation, thereby delaying the sharp drop of stress. Carbonation and brick aggregates may exert synergistic effects on modifying the descending branch; the modification effect induced by incorporating RBA is more remarkable for MRBAC subjected to deeper carbonation.

Comparative analysis of experimental data shows that carbonation imposes a significant influence on the shape of stress–strain curves of recycled brick-aggregate concrete under a constant RBA replacement ratio. As the RBA replacement ratio continuously increases, the discrepancies between the curves of specimens subjected to different carbonation durations and those of uncarbonated specimens with identical replacement ratios gradually diminish in terms of elastic-stage slope, strain position corresponding to peak stress, and attenuation trend of the post-peak descending branch. The curve profiles tend to converge. It can thus be concluded that RBA content is a key variable governing the magnitude of carbonation effect on stress–strain curves. A higher RBA replacement ratio corresponds to a milder influence of carbonation on curve characteristics.

#### 3.4.3. Peak Stress and Peak Strain

(1) Peak Stress

**Figure 13:**
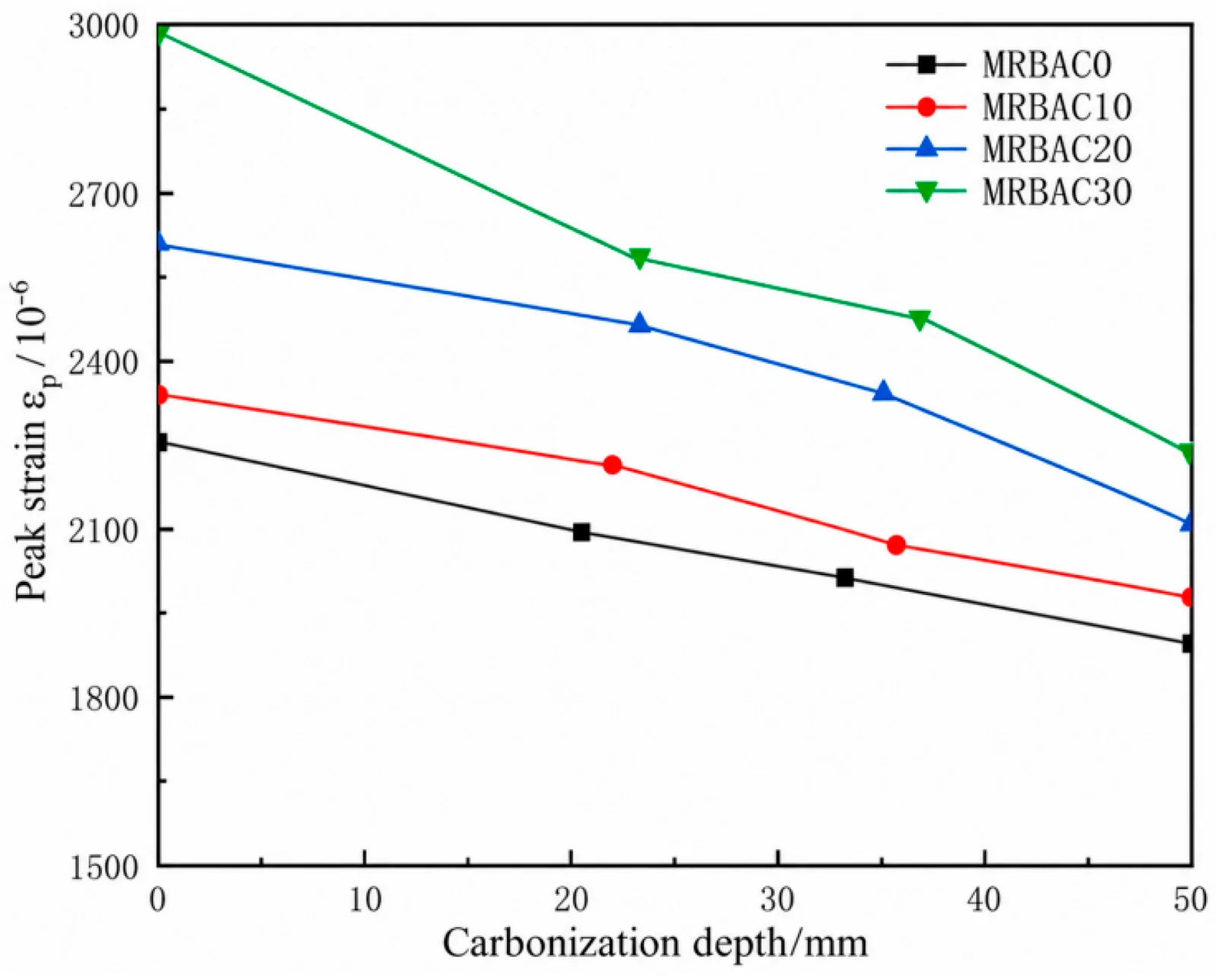
Variation in peak stress of MRBAC after carbonation.

The peak stress of MRBAC after carbonation is presented in Figure 13 and Table 9. It can be inferred that the mix proportion of coarse aggregates is the decisive factor for the initial strength of concrete. The peak stress of MRBAC decreases significantly with increasing RBA replacement ratio (the peak stress of MRBAC0 is 20% higher than that of MRBAC30). The rough surface and high water absorption of brick aggregates lead to insufficient hydration of surrounding cement paste and generate interfacial transition zones enriched with microcracks. Such regions constitute weak links prone to crushing under loading, forming a short-board effect in concrete mixed with multiple coarse aggregates. Nevertheless, the gap in peak stress gradually narrows with deepening carbonation. Except for specimens without RBA incorporation, the final peak stress of all other MRBAC specimens approaches 31 MPa, and fully carbonated specimens exhibit similar peak stress values. Carbonation provides a compensatory strengthening effect for concrete containing brick aggregates. When both factors act simultaneously on MRBAC under loading, they do not simply counteract each other but exhibit a dynamic compensation relationship. Carbonation products such as calcium carbonate preferentially fill the vulnerable regions inside concrete, including brick aggregates and their surrounding weak ITZs. Therefore, carbonation precisely strengthens the weakest zones within the concrete system, and the strengthening effect is particularly prominent for mixtures with high brick-aggregate content. In summary, carbonation significantly reduces the strength discrepancy among MRBAC mixtures with various RBA contents. From the perspective of long-term durability, carbonated MRBAC incorporating appropriate brick-aggregate content achieves superior peak stress compared with conventional recycled aggregate concrete.

**Figure 14:**
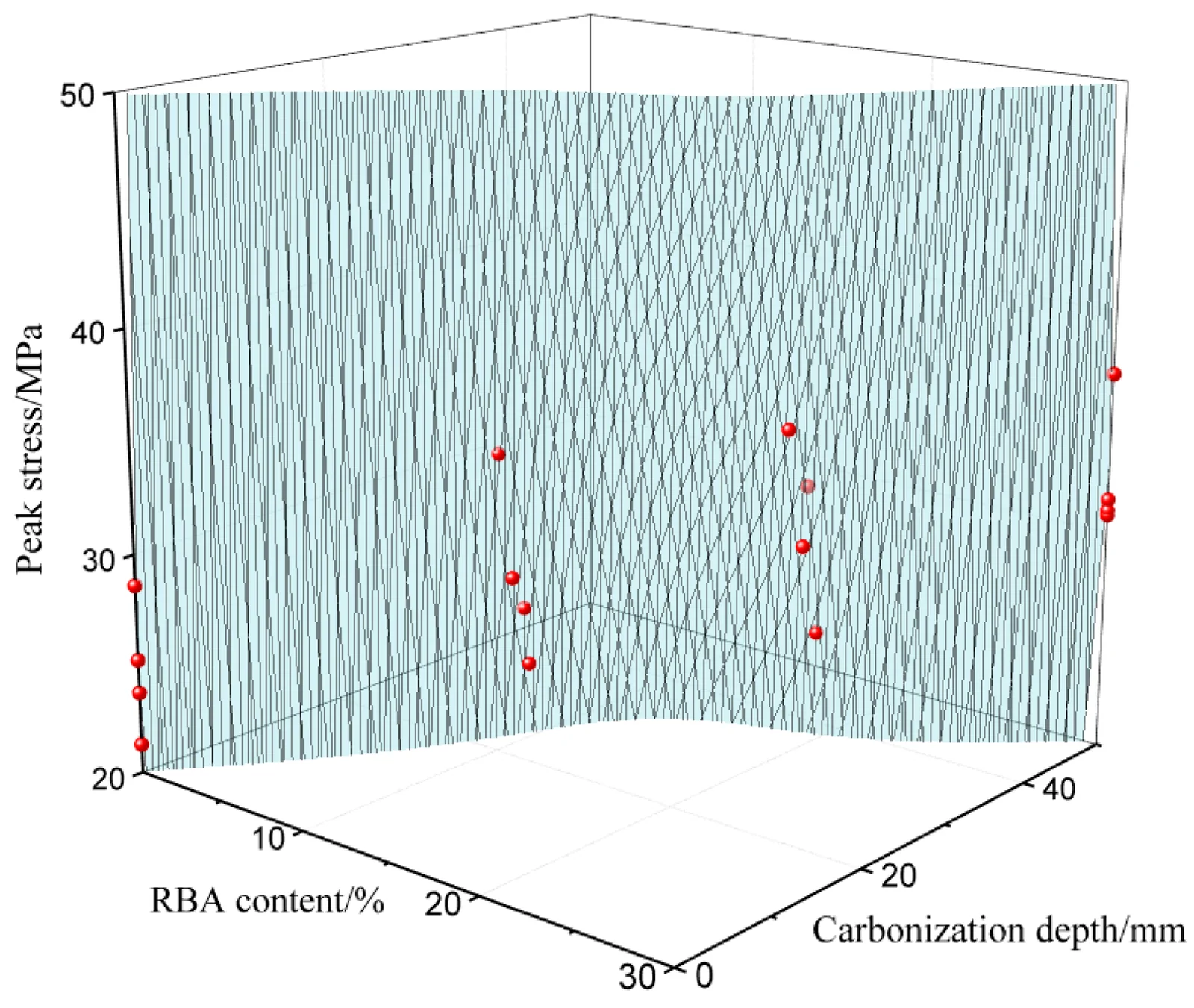
Fitting results of peak stress for carbonated MRBAC.

Using the measured experimental data, multivariate surface regression fitting for the peak stress of MRBAC is performed with carbonation depth and RBA content as independent variables. The fitted surface is shown in Figure 14.

The fitting expression is written as

(8) $$\begin{array}{c} \sigma_{p}=25.10r_{c}+31.98r_{c}^{2}-16.19r_{c}^{3}-1.76x_{c}+0.51x_{c}^{2}\\ R^{2}=0.999 \end{array}$$

where *σ*_p_ = peak stress (MPa); *r*_c_ = content of brick aggregate (%); *x*_c_ = carbonation depth (mm).

(2) Peak Strain

**Figure 15:**
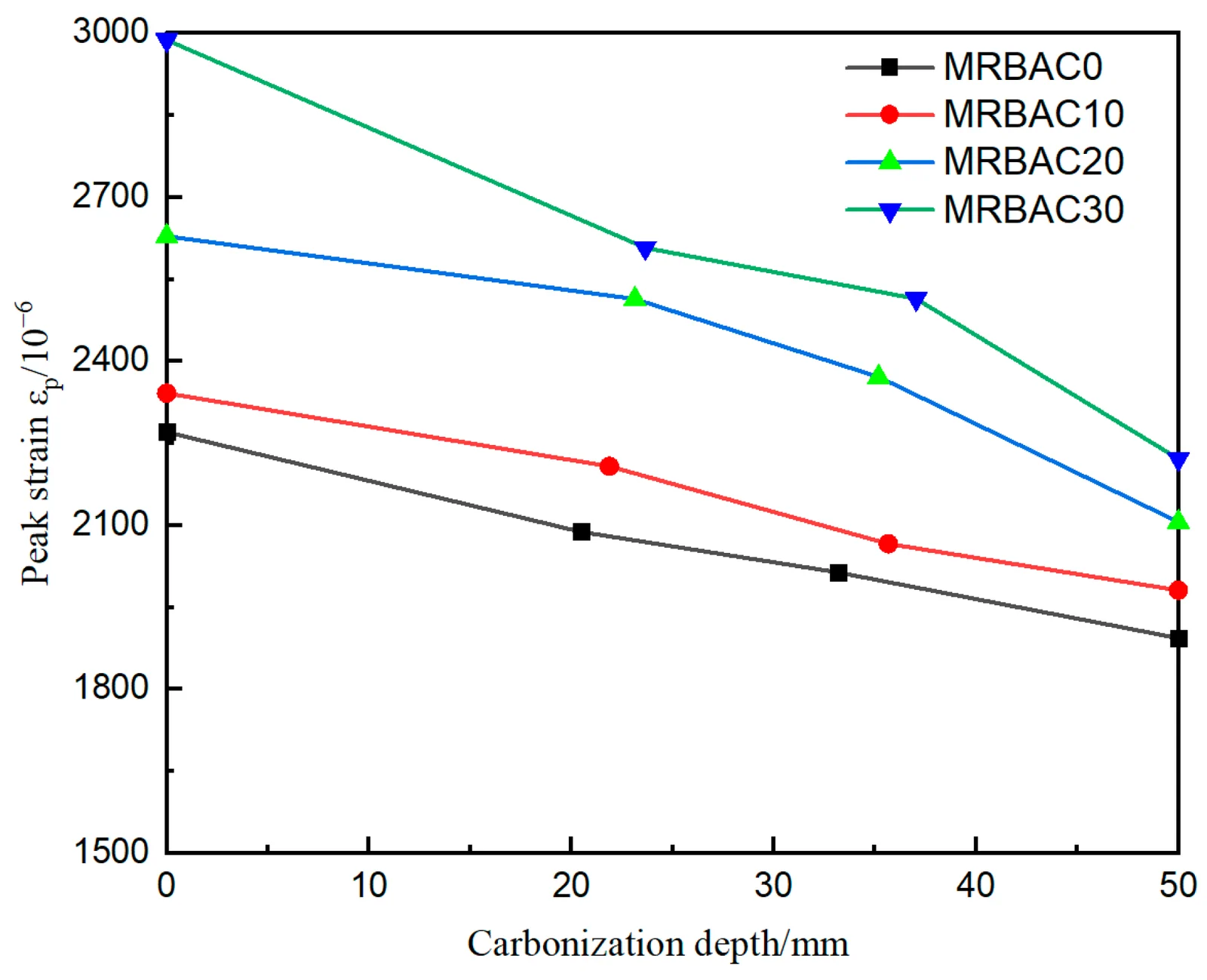
Variation in peak strain of MRBAC after carbonation.

The variation in peak strain of MRBAC after carbonation is illustrated in Figure 15 and Table 10. Test results indicate that peak strain rises noticeably with increasing RBA replacement ratio (the peak strain of MRBAC30 is 31.5% higher than that of MRBAC0). As carbonation depth increases, the axial deformation capacity of recycled coarse aggregate concrete specimens gradually declines. Carbonation products fill partial pores and densify the surface layer. However, such densification accompanied by non-uniform shrinkage generates micro-shrinkage stress inside concrete and weakens the ITZ between aggregates and cement paste. Consequently, carbonation densifies concrete yet renders it more brittle. It reduces the material’s capacity to undergo adaptive deformation via internal stress redistribution before failure, resulting in deteriorated deformability and more sudden failure. At low carbonation depth or in the uncarbonated state, RBA content directly determines the magnitude of peak strain. In the later stage of carbonation, the peak strain values of specimens with different RBA contents become closer (the difference in peak strain between MRBAC30 and MRBAC0 decreases from 31.5% to 17.3%). Carbonation weakens the plastic characteristics introduced by RBA. As demonstrated in Figure 15, the peak strain of MRBAC for all mix proportions declines with rising carbonation depth, and the reduction is more significant for mixtures with high brick-aggregate content.

**Figure 16:**
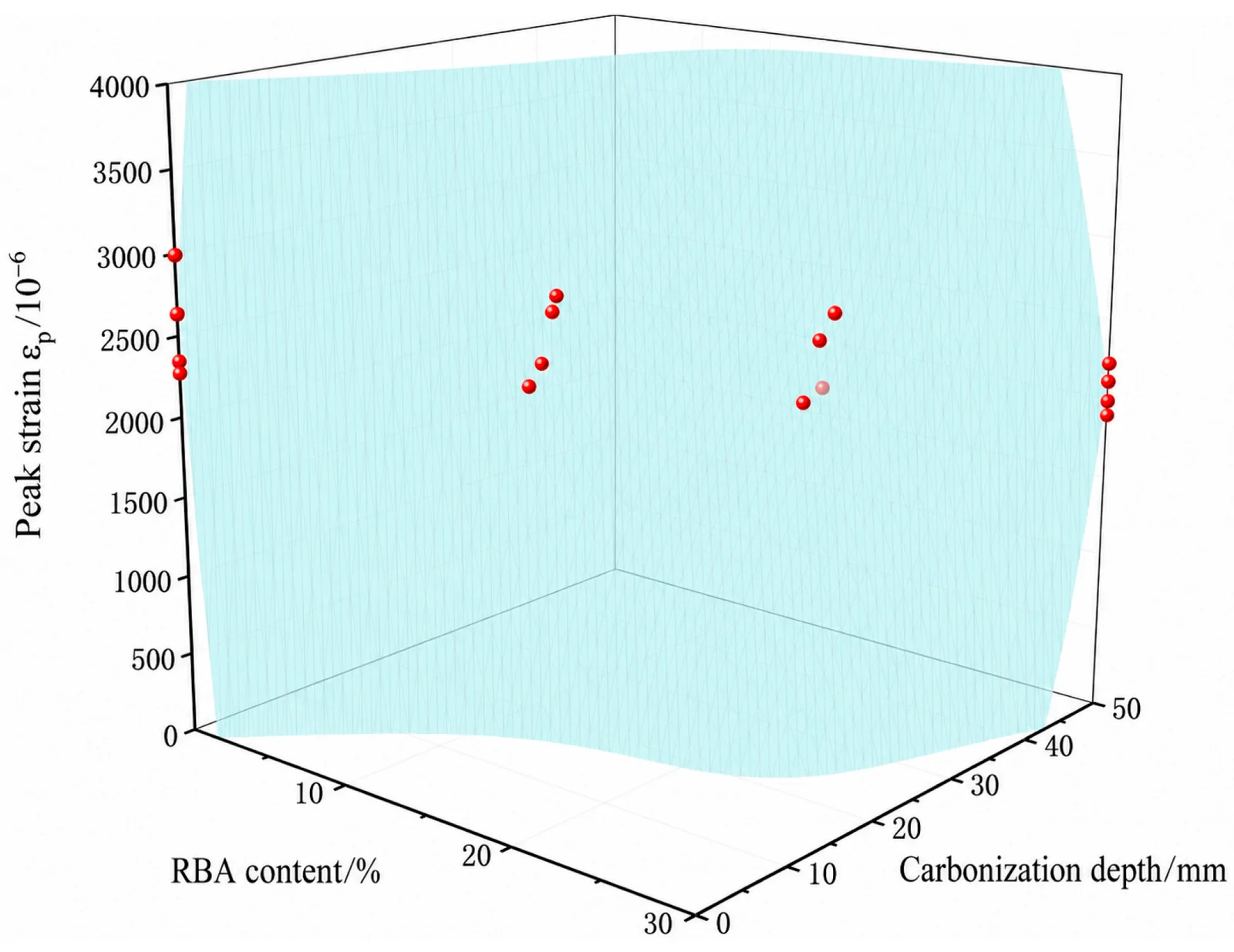
Fitting results of peak strain for carbonated MRBAC.

Based on experimental measurements, regression fitting for the peak strain of MRBAC is carried out with carbonation depth and RBA content as independent variables. The derived fitting formula is expressed as

(9) $$\begin{array}{c} \varepsilon_{p}=2556.95-1740.65r_{c}+838.22x_{c}+91.46r^{2}-23.67{x_{c}}^{2}-1.67r_{c}^{3}+0.25x_{c}^{3}\\ R^{2}=0.77 \end{array}$$

where *ε*_p_ = peak strain; *r*_c_ = content of brick aggregate (%); and *x*_c_ = carbonation depth (mm). The fitted surface is displayed in Figure 16.

The variation in peak strain is governed by two competing micro-factors. Carbonation-derived matrix densification reduces deformability and peak strain, while abundant micro-cracks and pores inside brick aggregates increase material deformability and peak strain. Such coupled effects lead to the observed trend in this work. Abbara et al. [14] also pointed out that mixed recycled aggregates significantly alter the peak-strain characteristics of concrete compared with ordinary concrete.

#### 3.4.4. Uniaxial Compressive Stress–Strain Model of MRBAC

In the research on high-strength recycled aggregate concrete, Luo [24] considered the influence of recycled aggregate replacement ratio on parameters for both ascending and descending branches.

(10) $$\begin{array}{l} y=ax+(3-2a)x^{2}+(a-2)x^{3},0\leq x<1\\ y=\frac{x}{b{\left(x-1\right)}^{2}+x},x\geq 1 \end{array}$$

In this study, the shape parameters in the recycled aggregate concrete stress–strain model proposed by Luo [24] are modified to establish the stress–strain model applicable to MRBAC.

**Figure 17:**
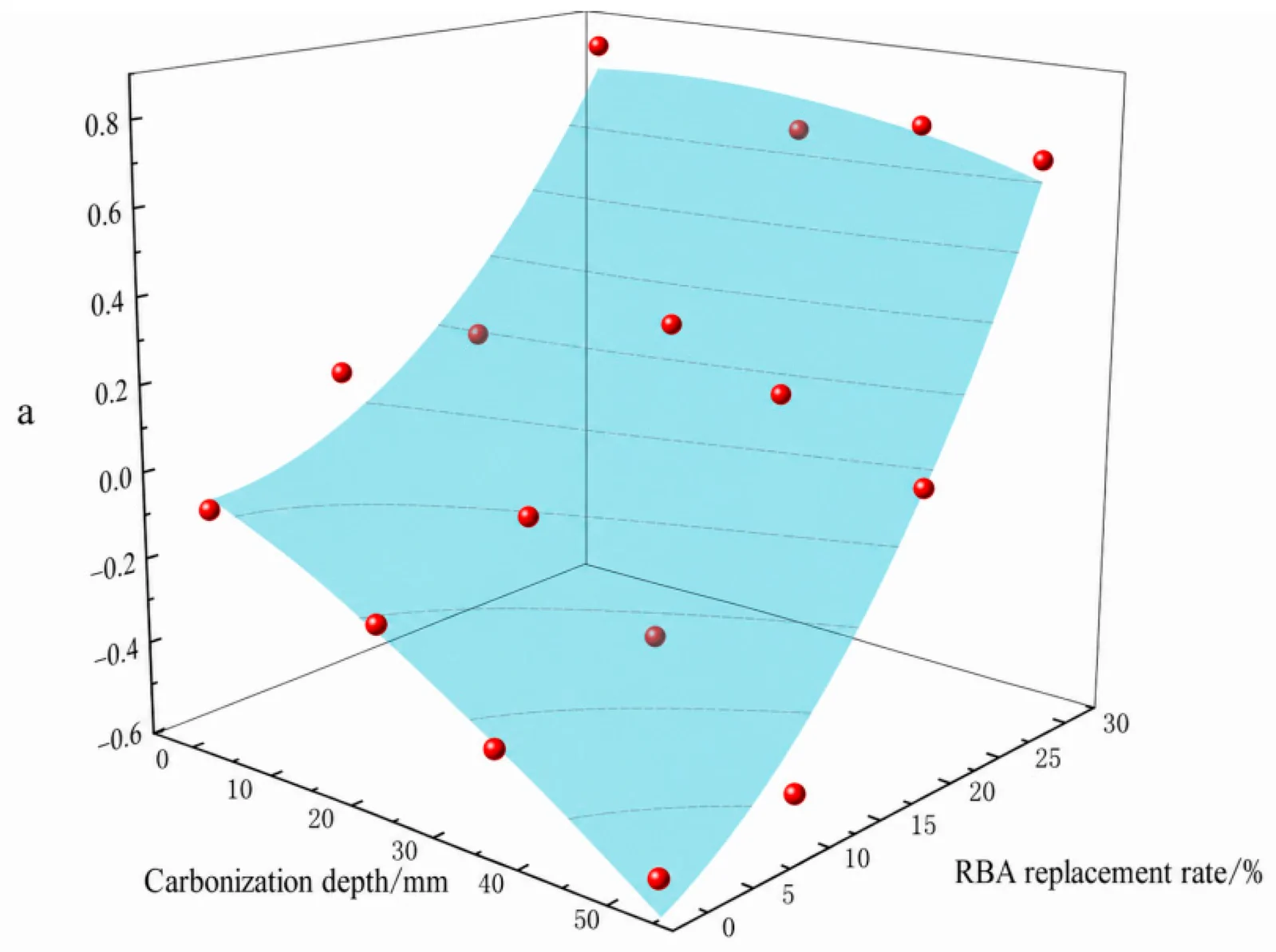
Fitting results of ascending branch shape parameter for carbonated MRBAC.

Regression analysis of the ascending branch shape parameter is conducted using carbonation depth and RBA replacement ratio as independent variables based on measured data. The fitting result of the ascending branch shape parameter *a* is shown in Figure 17, with the coefficient of determination R^2^ = 0.97. All regression and fitting analyses in this paper are performed using group-averaged experimental values.

(11) $$\begin{array}{l} a=-6.5\times 10^{-3}x_{c}-7.510^{-3}r_{c}-8.68\times 10^{-5}{x_{c}}^{2}+9.16\times 10^{-4}{r_{c}}^{2}+3.11\times 10^{-4}x_{c}r_{c}\\ R^{2}=0.975 \end{array}$$

where *a* = shape parameter of the ascending branch; *x*_c_ = carbonation depth (mm); and *r*_c_ = RBA replacement ratio (%).

**Figure 18:**
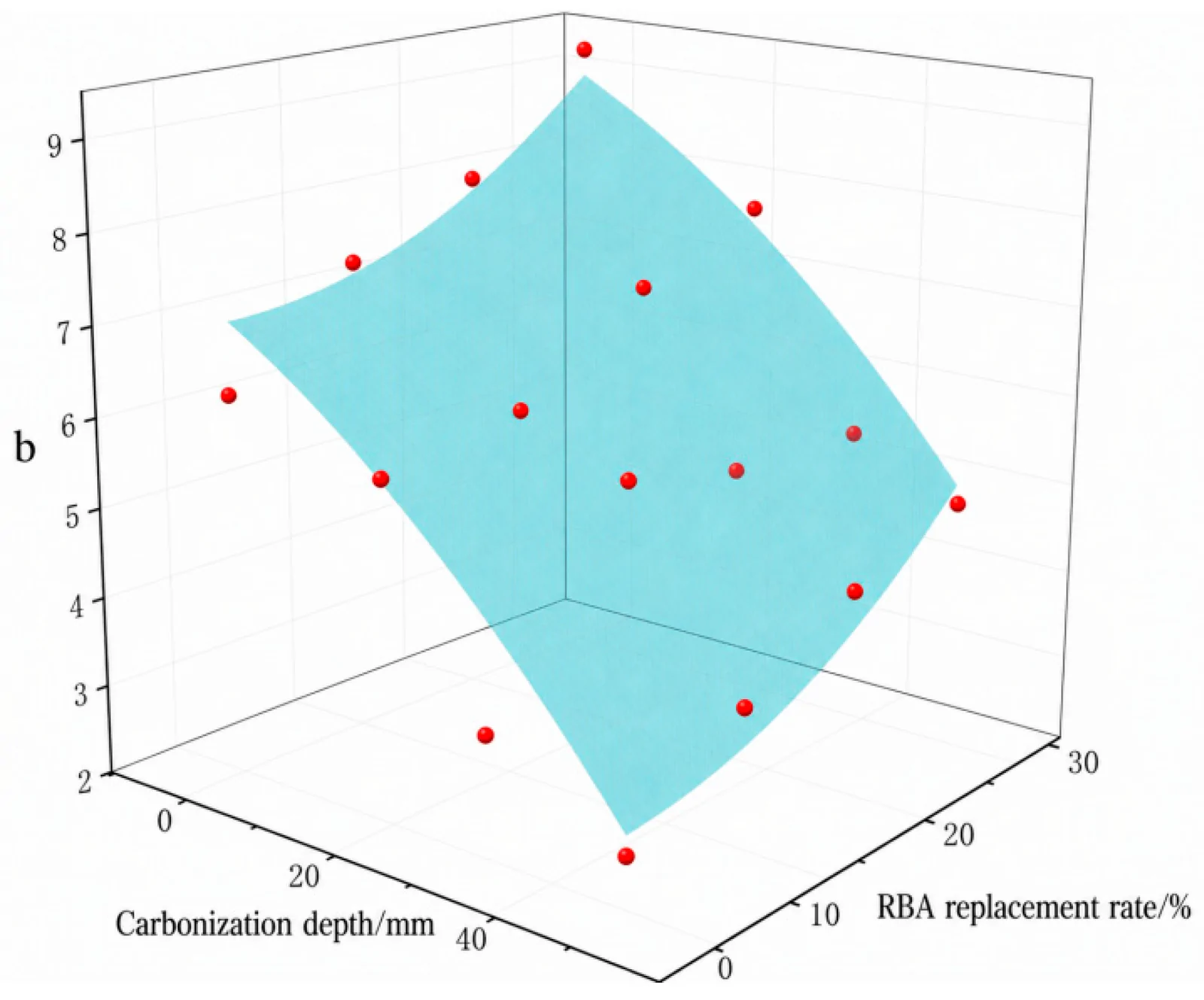
Fitting results of descending branch shape parameter for carbonated MRBAC.

Similarly, regression analysis of the descending branch shape parameter is performed taking carbonation depth and RBA replacement ratio as independent variables. The fitting result of the descending branch shape parameter *b* is presented in Figure 18.

(12) $$\begin{array}{l} b=7.05x_{c}-0.05r_{c}-6.87\times 10^{-4}{x_{c}}^{2}+2.15\times 10^{-3}{r_{c}}^{2}+2.42\times 10^{-4}x_{c}r_{c}\\ R^{2}=0.893 \end{array}$$

where *b* = shape parameter of the descending branch; *x*_c_ = carbonation depth (mm); *r*_c_ = RBA replacement ratio (%).

Based on the measured experimental data of MRBAC, Equations (10)–(12) were adopted to fit the ascending-branch shape parameter a and descending-branch shape parameter b. The fitted parameters and corresponding determination coefficients R^2^ are summarized in Table 11.

To verify the applicability of the proposed model, the computational model is compared with experimental results reported by other researchers. At present, limited studies are available on the stress–strain curves of carbonated MRBAC. Accordingly, the model established in this study is compared with the measured stress–strain curves of uncarbonated recycled aggregate concrete containing brick aggregates tested by Zhu [25], as well as the uniaxial compressive stress–strain constitutive formula for uncarbonated recycled brick-containing aggregate concrete proposed by Zhu [24]. The experimental program of Zhu [25] covered specimens with RBA contents ranging from 0% to 100%, and datasets with relatively low RBA replacement ratios are selected for comparison in this work.

Zhu [25] adopted recycled coarse aggregates originating from original C40 concrete with a water–cement ratio of 0.4. For specimens with 0% RBA content, the cubic compressive strength was remarkably higher than that of the specimens in the present test. When the RBA content reached 50%, the cubic compressive strength was close to that of the specimens in this study. Therefore, the MRBAC0 and MRBAC50 groups are selected for theoretical verification of the formula proposed herein.

**Figure 19:**
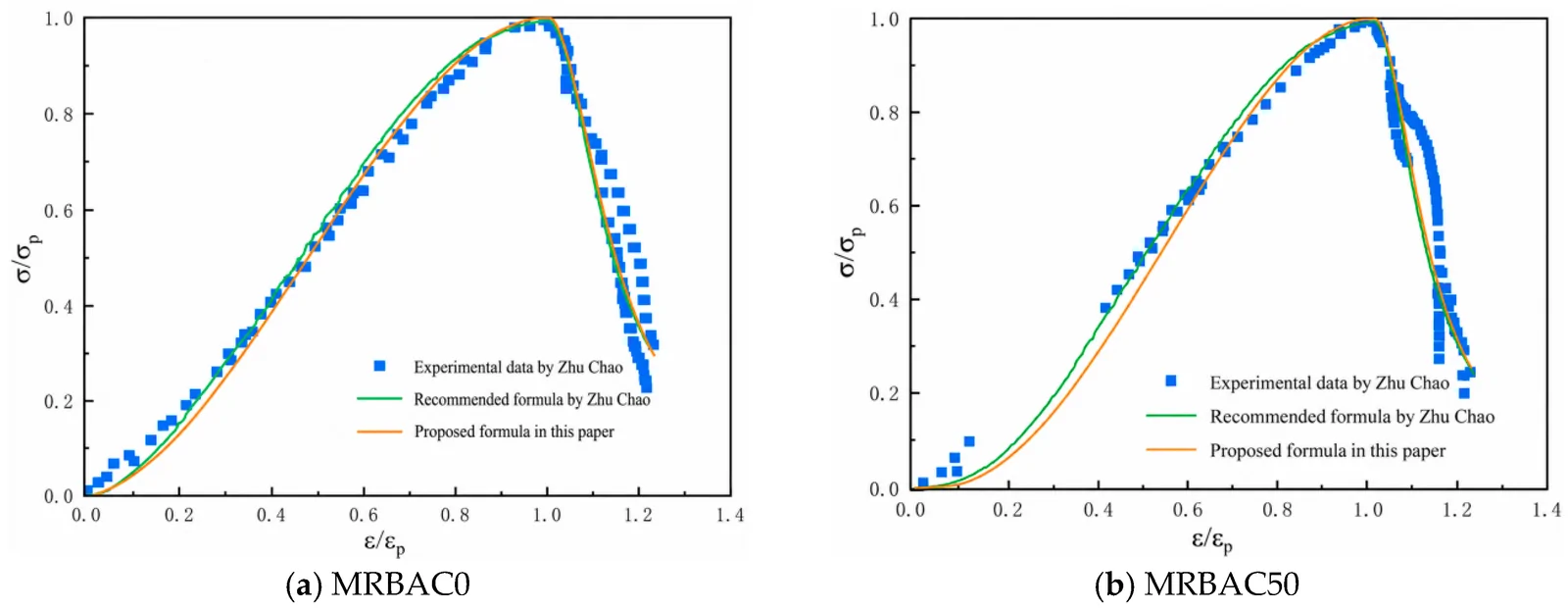
Comparison between experimental data and the formula proposed by Zhu [24] and in this study: (**a**) MRBAC0; (**b**) MRBAC50.

As illustrated in Figure 19, the descending branches predicted by the formula proposed in this study are similar to those from Zhu [25], showing good consistency in the post-peak region. In terms of the ascending branch, the fitting curve from Zhu [25] is fuller than that predicted by the model developed in this study, and this difference becomes more prominent with increasing RBA content. This can be attributed to the higher cubic compressive strength and elastic modulus of the specimens prepared by Zhu [25]. It is concluded that the proposed model exhibits better applicability for MRBAC with relatively low strength.

**Figure 20:**
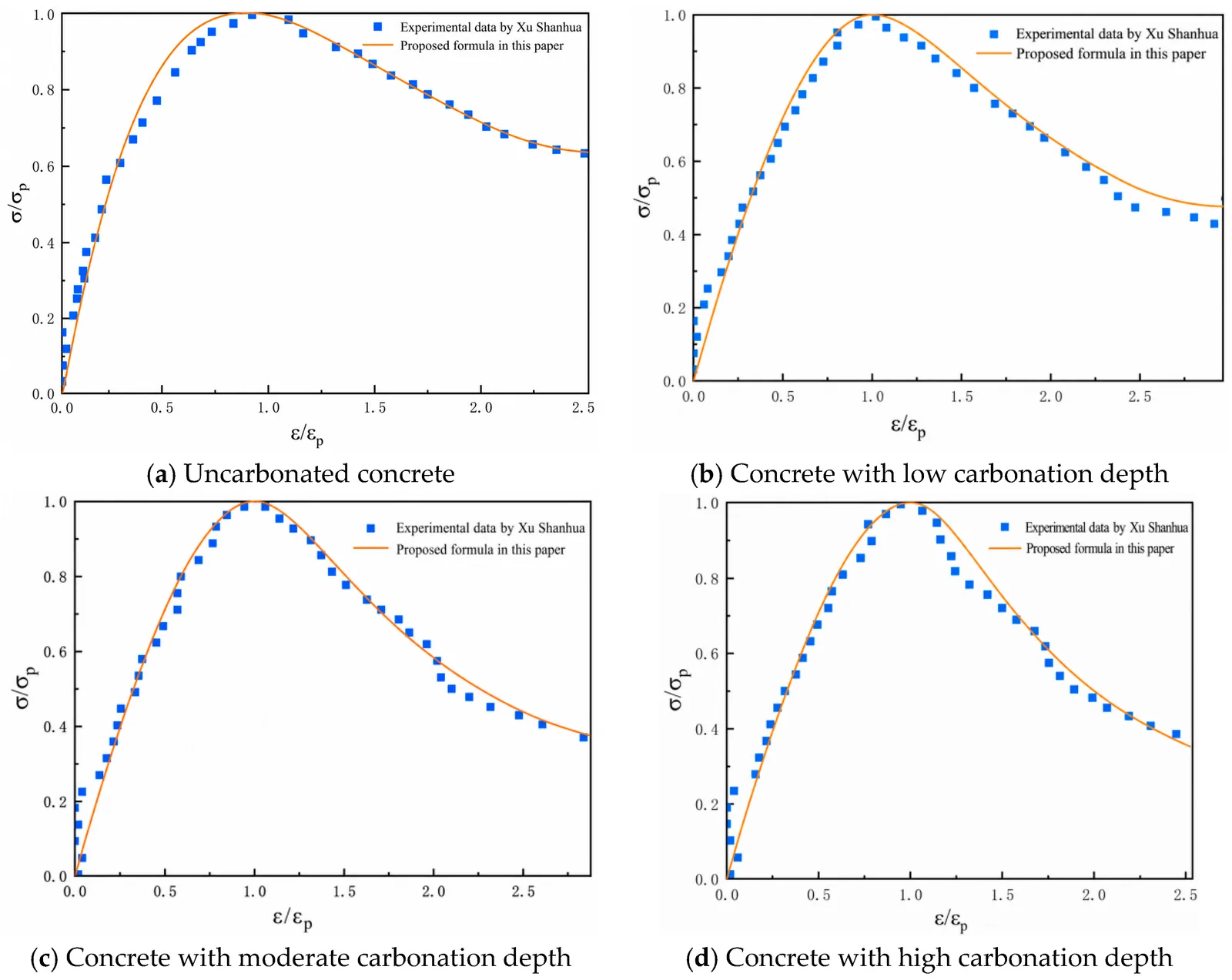
Comparison between experimental data from Xu [26] and the proposed formula: (**a**) Uncarbonated concrete; (**b**) Concrete with low carbonation depth; (**c**) Concrete with moderate carbonation depth; (**d**) Concrete with high carbonation depth.

To investigate the performance of the proposed model for carbonated recycled concrete, the formula developed in this work is compared with the uniaxial compressive stress–strain curves of concrete before and after carbonation reported by Xu [26]. Xu [26] conducted carbonation tests on concrete specimens and subsequently obtained corresponding stress–strain curves. In the research on the uniaxial compressive constitutive relationship of carbonated concrete under monotonic loading, Xu [26] classified concrete specimens into four groups according to different carbonation depths. The compressive strength of their specimens was below C30, which is comparable to the specimens in this study. Hence, their experimental data are compared with the uniaxial compressive stress–strain model proposed herein, as shown in Figure 20.

As shown in Figure 20, the proposed formula in this study can well fit the experimental data under most conditions. It exhibits satisfactory agreement with the measured data reported by Xu [26] at various carbonation depths, and the experimental data points distribute along the fitting curve. Only at relatively large carbonation depths and when ε/ε_p_ > 1.5, the curve predicted by the formula generally lies above the measured data points. This indicates that the proposed uniaxial compressive theoretical model for MRBAC may overestimate the ductility of carbonated recycled aggregate concrete when adopted for recycled concrete. This discrepancy is probably caused by the incorporation of brick aggregates in the specimens of this study, which yields higher ductility after carbonation compared with conventional recycled aggregate concrete. Overall, the proposed formula possesses good applicability for describing the stress–strain curves of concrete, especially for concrete ranging from the uncarbonated state to moderate carbonation.

## 4. Conclusions

In this paper, uniaxial compression tests were carried out on mixed recycled brick-aggregate concrete (MRBAC) subjected to accelerated carbonation, with recycled brick-aggregate (RBA) replacement ratio and carbonation duration as key variables. The coupled effects of RBA content and carbonation depth on carbonation resistance and mechanical behaviors were analyzed, and a phenomenological uniaxial compressive constitutive model for carbonated MRBAC was proposed. The main conclusions are drawn as follows:

(1) The cubic compressive strength of MRBAC decreases with higher RBA replacement ratios but rises continuously with extended carbonation exposure. After 120 d of full carbonation, cubic compressive strength gains range from 16.07% to 32.13%. Carbonation compensates for the strength loss induced by brick aggregates and reduces mechanical property discrepancies between mixtures with different RBA dosages.
(2) Elastic modulus increases monotonically with carbonation time, with more rapid stiffness enhancement in the early carbonation stage. Complete carbonation improves elastic modulus by 28.3–50.1%, while 30% RBA replacement reduces elastic modulus by approximately 38% relative to aggregate-free MRBAC.
(3) Carbonation significantly elevates peak compressive stress (average growth of 43.1% after full carbonation) and reduces peak strain (average reduction of 20.75%). RBA addition increases peak strain and softens post-peak descending curves, improving the ductility of MRBAC.
(4) A uniaxial compressive stress–strain constitutive model for carbonated MRBAC is proposed, which comprehensively considers the coupled effects of RBA replacement ratio and carbonation depth on curve shape coefficients. Independent validation against published experimental datasets confirms the model’s reliable applicability for low-strength mixed recycled brick-aggregate concrete under varying carbonation conditions. It should be noted that the empirical model in this study is valid for C25-grade MRBAC with a water–cement ratio of 0.52, RBA replacement ratio of 0–30%, and carbonation depth ranging from 0 mm to 50 mm. It is not recommended to directly extrapolate this model to parameters outside the above-mentioned ranges.

This study is based on laboratory accelerated-carbonation conditions. Further work can carry out natural carbonation exposure tests to verify model adaptability under real-service environments, explore mechanical behaviors of MRBAC with higher RBA replacement ratios (>30%), investigate multi-factor coupling effects such as carbonation–freeze–thaw cycles and conduct component-level tests to further calibrate and optimize the proposed constitutive model for structural application.

## Figures and Tables

**Table 1:** Physical and mechanical properties of cement.

Index	Loss on Ignition %	Specific Surface Area m^2^/kg	Initial Setting Time min	Final Setting Time min	3 d MPa		28 d MPa	
					Flexural Strength	Compressive Strength	Flexural Strength	Compressive Strength
Measured value	2.6	343	207	236	4.9	21.5	9.5	54.0
Standard limit	≤5.0	≥300	≥45	≤600	≥4.0	≥17.0	≥6.5	≥42.5

**Table 2:** Chemical composition of cement (mass fraction, %).

Component	SiO_2_	CaO	Al_2_O_3_	Fe_2_O_3_	MgO
Measured value	53.42	24.99	5.25	4.81	3.71

**Table 3:** Physical properties of recycled coarse aggregates.

Aggregate Type	Apparent Density kg/m^3^	Bulk Density kg/m^3^	Void Ratio %	Crushing Index %	Water Absorption %	Dust Content %
RCA	2466	1353	49	19.5	7.5	1.28
RBA	2271	1094	51	28.3	14.9	1.55

**Table 4:** Mix proportions of MRBAC.

Specimens	RBA Replacement Ratio %	Water–Cement Ratio	Cement kg/m^3^	Sand kg/m^3^	RCA kg/m^3^	RBA kg/m^3^	Water kg/m^3^
MRBAC0	0	0.52	400	550	1200	0	208
MRBAC10	10	0.52	400	550	1080	120	208
MRBAC20	20	0.52	400	550	960	240	208
MRBAC30	30	0.52	400	550	840	360	208

**Table 5:** Specimen arrangement.

Test Item	Specimen Size mm	RBA Replacement Ratio %	Carbonation Age d	Total Specimen Quantity
Cubic compressive strength	100 × 100 × 100	0, 10, 20, 30	0, 40, 80, 120	96
Carbonation depth measurement	100 × 100 × 100	0, 10, 20, 30	0, 40, 80, 120	96
Uniaxial compression & constitutive relationship	100 × 100 × 300	0, 10, 20, 30	0, 40, 80, 120	48

**Table 6:** Carbonation depths.

Carbonation Time d	Carbonation Depth mm			
	MRBAC0	MRBAC10	MRBAC20	MRBAC30
**0**	0	0	0	0
**40**	20.46	21.88	23.14	25.65
**80**	33.2	35.66	35.18	37.04
**120**	50	50	50	50

**Table 7:** Cubic compressive strength of MRBAC after carbonation.

Carbonation Time d	Cubic Compressive Strength MPa			
	MRBAC0	MRBAC10	MRBAC20	MRBAC30
0	32.35	30.23	29.04	28.2
40	33.74	32.37	31.56	30.34
80	36.26	34.57	33.54	29.27
120	37.55	38.92	38.37	35.6

**Table 8:** Normalized elastic modulus *E*_c_/*E*_cp_ of MRBAC after carbonation.

Carbonation Time d	Normalized Elastic Modulus *E*_c_/*E*_cp_			
	MRBAC0	MRBAC10	MRBAC20	MRBAC30
0	0.77673	0.73541	0.66803	0.67556
40	0.86792	0.80156	0.71721	0.71556
80	0.95283	0.93774	0.78689	0.8
120	1	1	1	1

**Table 9:** Peak stress for carbonated MRBAC.

RBA Content %	Carbonation Depth mm	Measured Peak Stress MPa
0	0	28.6
10	20.46	33.7
20	33.2	35
30	50	37.2
0	0	25.2
10	21.88	27.8
20	35.66	32.1
30	50	31.5
0	0	23.7
10	23.14	26.2
20	35.18	29.4
30	50	30.8
0	0	21.3
10	23.65	23.5
20	37.04	25.1
30	50	31

**Table 10:** Peak strain for carbonated MRBAC.

RBA Content %	Carbonation Depth mm	Measured Peak Strain 10^−6^
0	0	2270.7
10	20.46	2088.2
20	33.2	2013.6
30	50	1893.5
0	0	2341.5
10	21.88	2207.7
20	35.66	2066.2
30	50	1981.3
0	0	2628.3
10	23.14	2513.8
20	35.18	2371.2
30	50	2105.8
0	0	2987.3
10	23.65	2607.2
20	37.04	2514.1
30	50	2221.4

**Table 11:** Fitted parameters for carbonated MRBAC.

RBA Content %	Carbonation Time d	a	b	R^2^
0	0	−0.09	6.25	0.984
0	40	−0.23	5.76	0.995
0	80	−0.42	5.32	0.975
0	120	−0.58	2.51	0.992
10	0	0.14	7.38	0.973
10	40	−0.09	7.12	0.985
10	80	−0.28	4.63	0.970
10	120	−0.54	3.51	0.972
20	0	0.15	8.04	0.935
20	40	0.27	7.14	0.939
20	80	0.16	2.31	0.962
20	120	0.02	3.28	0.949
30	0	0.8116	9.27	0.995
30	40	0.6686	7.74	0.961
30	80	0.72	5.36	0.964
30	120	0.68	4.81	0.988

## Data Availability

The original contributions presented in this study are included in the article. Further inquiries can be directed to the corresponding authors.
